# Electrolyte Additive-Assembled Interconnecting Molecules–Zinc Anode Interface for Zinc-Ion Hybrid Supercapacitors

**DOI:** 10.1007/s40820-025-01794-1

**Published:** 2025-05-21

**Authors:** Yang Li, Xu Li, Xinya Peng, Xinyu Yang, Feiyu Kang, Liubing Dong

**Affiliations:** 1 School of Materials and Energy, Foshan University, Foshan, 528000 People’s Republic of China; 2 Tsinghua Shenzhen International Graduate School, Tsinghua University, Shenzhen, 518055 People’s Republic of China; 3 College of Chemistry and Materials Science, Jinan University, Guangzhou, 511443 People’s Republic of China

**Keywords:** Zinc-ion hybrid supercapacitor, Zinc anode, Electrolyte additive, Cycling stability, Electrochemical kinetics

## Abstract

Zinc-ion hybrid supercapacitors (ZHSs) are promising energy storage systems integrating high energy density and high-power density, whereas they are plagued by the poor electrochemical stability and inferior kinetics of zinc anodes. Herein, we report an electrolyte additive-assembled interconnecting molecules–zinc anode interface, realizing highly stable and fast-kinetics zinc anodes for ZHSs. The sulfobutyl groups-grafted β-cyclodextrin (SC) supramolecules as a trace additive in ZnSO_4_ electrolytes not only adsorb on zinc anodes but also self-assemble into an interconnecting molecule interface benefiting from the mutual attraction between the electron-rich sulfobutyl group and the electron-poor cavity of the adjacent SC supramolecule. The interconnecting molecules–zinc anode interface provides abundant anion-trapping cavities and zincophilic groups to enhance Zn^2+^ transference number and homogenize Zn^2+^ deposition sites, and meanwhile, it accelerates the desolvation of hydrated Zn^2+^ to improve zinc deposition kinetics and inhibit active water molecules from inducing parasitic reactions at the zinc deposition interface, making zinc anodes present superior reversibility with 99.7% Coulombic efficiency, ~ 30 times increase in operation lifetime and an outstanding cumulative capacity at large current densities. ZHSs with 20,000-cycle life and optimized rate capability are thereby achieved. This work provides an inspiring strategy for designing zinc anode interfaces to promote the development of ZHSs.

**Supplementary Information:**

The online version contains supplementary material available at 10.1007/s40820-025-01794-1.

## Highlights

- An important topic about zinc-ion hybrid supercapacitors is proposed, i.e., how to achieve not only ultrastable but also fast-kinetics zinc anodes to match capacitive cathodes featuring ultralong cycle life and fast charge storage ability.
- An electrolyte additive-assembled interconnecting molecules–zinc anode interface is proposed, and its regulation mechanisms on zinc plating/stripping behaviors are revealed.
- A scalable strategy is reported for achieving highly stable and fast-rate zinc anodes toward zinc-based electrochemical energy storage systems.

**Supplementary Information:**

The online version contains supplementary material available at 10.1007/s40820-025-01794-1.

## Graphical abstract

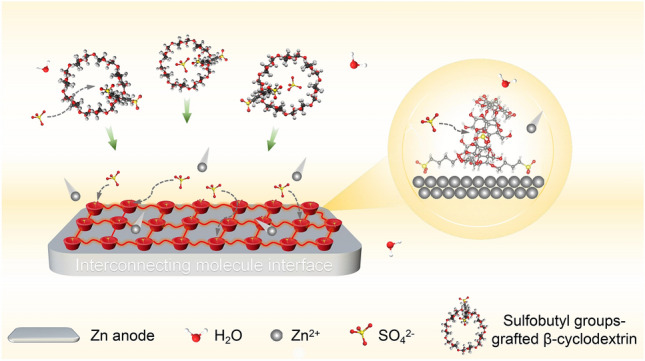

## Introduction

The urgent problems of energy shortage and environmental pollution are driving new technologies in the field of energy storage. Aqueous zinc-ion hybrid supercapacitors (ZHSs) theoretically combine the advantages of batteries and supercapacitors, presenting the potential to realize extreme safety, high energy density, fast charge/discharge rate and ultralong lifespan [1, 2]. Therefore, zinc-based ZHSs have been regarded as a competitive candidate for next-generation energy storage systems. Although applying zinc metal anodes with a large theoretical capacity (820 mAh g^−1^ and 5855 Ah L^−1^) and low redox potential (-0.763 V *vs*. standard hydrogen electrode) is conducive to improving the energy density of ZHSs [3, 4], the electrochemical instability and sluggish kinetics make zinc anodes hard to match capacitive cathodes (*e.g.*, porous carbon) with ultralong cycle life and fast charge storage/release ability in ZHSs. To be specific, the thermodynamically favorable form of zinc ions in aqueous zinc-salt electrolytes is hydrated ions, such as [Zn(H_2_O)_n_]^2+^ (*n* = 5–6) in aqueous 2 M ZnSO_4_ solution which is a widely used electrolyte for ZHSs. The desolvation of hydrated zinc ions at the electrolyte–zinc anode interface has to overcome a high energy barrier, thus limiting the electrochemical kinetics of zinc anodes [5, 6], and meanwhile, the free water molecules desolvated from the solvation shell of the hydrated zinc ions not only corrode metallic zinc anodes to generate electrically insulated zinc hydroxides but also are easily reduced to hydrogen gas [7–9]. The hydrogen evolution and corrosion reactions disrupt both the electric field and ion field of the zinc deposition interface, causing inhomogeneous zinc deposition and finial zinc dendrites [10, 11]. In addition, zinc electrodeposition starts with the generation of scattered zinc nuclei at the zinc deposition interface, while the newly formed zinc nucleus with a large curvature radius and high surface energy creates the “tip effect” and spontaneously attracts zinc ions to laterally diffuse to deposit on them [12, 13], which is also an important factor inducing the formation of dendrites. As a result, the dendrite growth, parasitic reaction and high desolvation energy barrier issues result in inferior electrochemical stability and sluggish kinetics of zinc anodes, making it impossible to realize ultralong-life and high-rate ZHSs.

To solve the above-mentioned dendrite growth and parasitic reaction issues of zinc anodes, considerable efforts have been dedicated to manipulating electrolyte solvation structure and regulating zinc deposition interface chemistry to homogenize the ion concentration field at the zinc deposition interface and prevent free water molecules from entering into the zinc deposition interface, thus restraining hydrogen evolution, corrosion reactions and dendrite growth. Various zinc anode stabilization strategies including optimizing zinc anode structure [14–17], constructing artificial interfaces [3, 18–22], designing functional separators [23–26] and regulating electrolyte composition [27–32], have been proposed. Unfortunately, some strategies inhibiting dendrite growth by slowing down zinc-ion transport at the zinc deposition interface pursue long-term stability of zinc anodes at the expense of fast kinetics, which are not applicable for ZHSs that are required to possess outstanding power density. Moreover, the electrolyte regulation strategy of adding a small amount of organic molecule additives into conventional zinc-salt solutions presents good compatibility with the scalable production of ZHSs since it inherits the merits of aqueous zinc-salt solutions such as high ionic conductivity, nonflammability and low cost [33–35]. Such a strategy stabilizes the zinc deposition interface through organic molecule additives spontaneously adsorbing on zinc anodes to homogenize zinc-ion flux and block water molecules. However, hydrated zinc ions moving under the action of the electric field push weakly interacting organic molecules away, which provides a channel for active water molecules and anions to enter into the zinc deposition interface to launch parasitic reactions and dendrite growth, challenging the realization of ultralong-life zinc anodes required for ZHSs.

Herein, an interconnecting molecule–zinc anode interface was self-assembled by introducing sulfobutyl groups-grafted β-cyclodextrin (SC) supramolecules as a trace additive into ZnSO_4_ electrolytes, realizing highly stable and fast-kinetics zinc anodes for ZHSs. The SC supramolecules would not only remodel the Zn^2+^ solvation structure and break the continuous H-bond network in the bulk electrolyte but also spontaneously adsorb on zinc anodes to self-assemble into an interconnecting molecules–zinc anode interface through the mutual attraction between the electron-rich sulfobutyl groups and the electron-poor cavity of the adjacent SC supramolecule. As a result, zinc anodes in the ZnSO_4_-based electrolyte with a rationalized SC concentration were endowed with superior reversibility, significantly extended lifetimes and optimized rate performance. The regulation mechanisms on the zinc plating/stripping behaviors of the SC supramolecule additive were revealed based on experimental analysis and theoretical computations. Especially, the interconnecting molecules–zinc anode interface provided abundant anion-trapping cavities and zincophilic groups to enhance Zn^2+^ transference number and homogenize Zn^2+^ deposition sites, and meanwhile, it accelerated the desolvation of Zn^2+^ to promote zinc deposition kinetics and inhibit active water from inducing parasitic reactions at the zinc deposition interface. Further, ultralong-life and high-rate ZHSs were enabled by the SC additive-assembled interconnecting molecules–zinc anode interface.

## Experimental Section

### Materials

Zinc sulfate heptahydrate (ZnSO_4_·7H_2_O, AR), sulfobutylether-β-cyclodextrin sodium salt (SC, ≥ 98%), β-cyclodextrin (β-CD, 96%) and citric acid monohydrate (99.5%) were purchased from Shanghai Aladdin Bio-Chem Technology Co., Ltd. Vanadium pentoxide (V_2_O_5_, 99.99%) was produced by Shanghai Macklin Biochemical Co., Ltd. Activated carbon (AC, model: XFP01) powder was obtained from Nanjing XFNANO Tech Co., Ltd. All chemicals were used without further purification. Zinc foils with a thickness of 50 µm were supplied by Shanghai Weidi Metal Material Technology Co., Ltd.

### Preparation of Electrolytes and Electrodes

ZnSO_4_·7H_2_O powder and SC powder were dissolved in deionized water to prepare aqueous ZnSO_4_-SC hybrid electrolytes, in which the molar concentration of the ZnSO_4_ and the SC additive was 2 M and *x* mM, respectively, and the hybrid electrolytes were denoted as ZSO/SC-*x*. Pure 2 M ZnSO_4_ aqueous electrolyte was prepared without the addition of the SC. The AC cathodes were fabricated by coating a homogenous slurry composed of the AC powder, acetylene black conductive additive, polyvinylidene fluoride binder and N-methyl-2-pyrrolidone solvent onto a stainless steel current collector and vacuum-drying at 80 °C completely. VO_2_ powder synthesized through the method reported in the literature [18] was made into the VO_2_ cathodes according to the preparation procedures of the AC cathodes. The mass loading of the active materials in the aforementioned cathodes is 1.5–2.0 mg cm^−2^.

### Material Characterizations

The micromorphology of the materials and the electrodes was observed using scanning electron microscopy (SEM, Zeiss Supra55) and a 3D laser scanning confocal microscopy (LSCM, KEYENCE VK-X150). The phase composition of the samples was studied through X-ray diffraction (XRD) tests on a diffractometer equipped with Cu Kα radiation (Rigaku MiniFlex 600). The electrolyte solvation structure and the functional groups of the samples were analyzed through Raman spectra on a laser confocal Raman spectrometer (LabRAM HR Evolution with a laser excitation of 532 nm) and Fourier transform infrared (FTIR) spectra on a PerkinElmer Spectrum Two infrared instrument. A conductivity meter (Mettler Toledo, SevenCompact S230), a pH meter (Mettler Toledo, FiveEasy Plus FE28) and a viscosimeter (NDJ-8S) were applied to determine the ionic conductivity, the pH value and the viscosity of the electrolytes, respectively. Each test was repeated at least three times, and the standard deviations of the measured ionic conductivity, pH value and viscosity were smaller than 0.17 mS cm^−1^, 0.05 and 0.05 mPa s, respectively, for all samples. Contact angle tests were performed on a contact angle meter (DSA-11), and the test was repeated three times for each sample to guarantee the reliability of the data. The standard deviation of the obtained contact angle values was 1.1°, 0.5°, 0.8°, 1.2°, 0.4° and 0.5°, respectively, for the ZnSO_4_, ZSO/SC-5, ZSO/SC-10, ZSO/SC-20, ZSO/SC-30 and ZSO/SC-50 electrolytes. Then the wetting free energy of the electrolytes was calculated based on Eq. (1):

1 $$ \Delta G = \frac{RT}{3}\ln \frac{{(1 - \cos \theta )^{2} (2 + \cos \theta )}}{4} $$

where ∆*G*, *T* and *θ* are the wetting free energy, the testing temperature and the contact angle, respectively, and R is the ideal gas constant of 8.314 J mol^−1^ K^−1^. A metallurgical microscope (6XB-PC) was used to record the zinc deposition behaviors in real time in a transparent cell with a Zn//Zn symmetric configuration, and the current density was 5 mA cm^−2^.

### Electrochemical Measurements

Zn//Zn symmetric cells and Cu//Zn asymmetric cells were assembled, in which zinc foil, copper foil and glass fiber membrane (260 μm in thickness) served as the zinc electrode, Cu electrode and separator, respectively. The galvanostatic charge/discharge (GCD) technique was used to evaluate the electrochemical stability and rate performance of the zinc anodes in the Zn//Zn symmetric cells under various areal current densities and zinc plating/stripping capacities. The chronoamperometry (CA) curve of the zinc deposition process was acquired by imposing a -150 mV overpotential on the Zn//Zn symmetric cells. The Tafel plot was obtained by using the linear sweep voltammetry method with a scan rate of 10 mV s^−1^. The electric double-layer capacitance (EDLC) of the electrolyte–anode interface was determined through cyclic voltammetry (CV) tests at 10 mV s^−1^ of the Zn//Zn symmetric cells and calculated using Eq. (2):

2 $$ EDLC = i_{c} /\nu = \frac{{i_{{ + ,0{\text{V}}}} - i_{{ - ,0{\text{V}}}} }}{2 \times \nu } $$

in which ν is the scan rate, and *i*_+,0 V_ (*i*_−,0 V_) is the response current at 0 V during the positive (negative) scanning process. The transference number of Zn^2+^ in different electrolytes was measured through in-order electrochemical impedance spectroscopy (EIS)-CA-EIS tests of the assembled Zn//Zn symmetric cells, during which an amplitude of 5 mV and frequency range of 0.1 Hz to 100 kHz were applied for the EIS tests and a polarization potential of -25 mV was applied for the CA test. Detailed calculation of the Zn^2+^ transference number (*t*_+_) follows Eq. (3):

3 $$ t_{ + } = \frac{{I_{{\text{s}}} \left( {\Delta V - R_{0} I_{0} } \right)}}{{I_{{\text{s}}} (\Delta V - R_{s} I_{s} )}} $$

where Δ*V* is the polarization potential of -10 mV. *R*_0_ and *R*_s_ are the resistance obtained from the EIS tests before and after the CA test, respectively, and *I*_0_ and *I*_s_ are the initial and steady-state currents during the CA test, respectively. The zinc plating/stripping Coulombic efficiency in different electrolytes was studied by discharging the Cu//Zn asymmetric cells to deposit zinc on the Cu electrode and then charging the asymmetric cells to 0.5 V as the cutoff voltage. Coin cell-typed Zn//AC ZHSs and Zn//VO_2_ zinc-ion batteries (ZIBs) were assembled. Their CV curves and EIS spectra were recorded on an electrochemical workstation (Bio-Logic VSP-300), and GCD tests were implemented on a battery testing instrument (LAND CT2001A). Besides, pouch cell-typed ZHSs with the ZSO/SC-10 electrolyte were constructed by sandwiching a glass fiber separator between the zinc foil anode and the AC cathode and then being sealed in an aluminum plastic film, in which the mass of the AC active material reached 23.6 mg.

### Computational Methods

The density functional theory (DFT) calculations were implemented by the Vienna Ab initio Simulation Package (*i.e.*, VASP), in which the interactions of the valence and core electrons were expressed by the projector augmented-wave method. The exchange–correlation energy was approximately described by Perdew–Burke–Ernzerhof (PBE) functional based on the generalized gradient approximation. The plane-wave cutoff energy was set to 500 eV, and the Gamma-centered *k*-point mesh was applied in the first Brillouin zone. The Zn(002) slab was built with six atomic layers in a hexagonal 7 × 7 supercell with the bottom two layers fixing during structural relaxation, and the convergence threshold and maximum force were set to 10^–4^ eV for energy and 0.02 eV Å^−1^ for ionic relaxation. All the structures were produced by the Visualization for Electronic and Structural Analysis (VESTA) software. The adsorption energy (*E*_ads_) between the zinc slab and different adsorbates was calculated as follows:

4 $$ E_{{{\text{ads}}}} = E_{{{\text{total}}}} - E_{{{\text{Zn}}(002)}} - E_{{{\text{adsorbate}}}} $$

where *E*_total_, *E*_Zn(002)_ and *E*_adsorbate_ are the total energy of the optimized model for the adsorbate on the Zn(002) surface, the energy of Zn(002) surface and the energy of the adsorbate, respectively. Similarly, the binding energy between two components was defined as the energy difference between their combined state and free state. In addition, molecular dynamics (MD) simulations were performed using the Groningen Machine for Chemical Simulations (GROMACS) package. The forced field parameters were obtained from Amber force fields. The H_2_O molecule was simulated with a simple point charge model. The size of the box was 5.51 × 5.51 × 5.51 nm^3^, and periodic boundary conditions were set in all three directions. The amounts of H_2_O, ZnSO_4_ and SC supramolecules in the simulation cells were 5386, 200 and 1, respectively, which were consistent with the molar ratios of these species in the aqueous ZSO/SC-10 hybrid electrolyte. The electrostatic interactions were computed using the particle-mesh Ewald (PME) method. A cutoff length of 1.0 nm was used during the calculation of electrostatic interactions and nonelectrostatic interactions in real space, and the integration time step was 1.0 fs. The system was annealed from 0 to 298 K over a period of 0.5 ns, followed by running for another 2.0 ns to reach equilibrium. A 10-ns NPT simulation was run for post-processing analysis.

## Results and Discussion

**Fig. 1:**
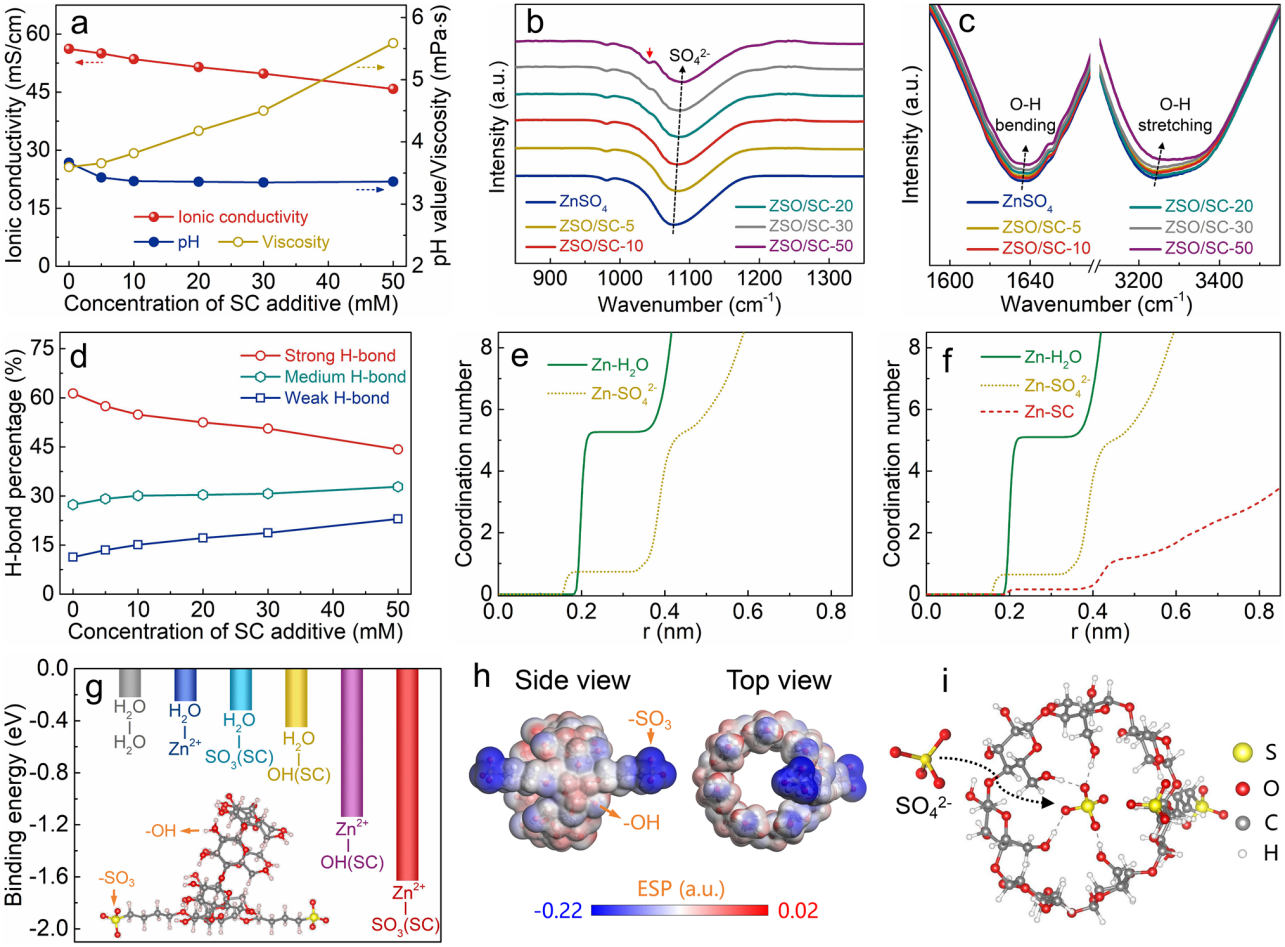
**a** Physical properties of the ZnSO_4_-SC hybrid electrolytes. FTIR spectra of the hybrid electrolytes: **b** stretching vibration signal of SO_4_^2−^ and **c** bending vibration and stretching vibration signals of O–H. **d** H-bonding state of the hybrid electrolytes. MD simulations on the coordination structure of Zn^2+^ in **e** ZnSO_4_ and **f** ZSO/SC-10 electrolytes. DFT calculation results: **g** binding energy between various species in the hybrid electrolytes (inset: molecule structure of the SC); **h** electrostatic potential distribution of the SC supramolecule in side and top views; **i** thermodynamically favorable model of the SC supramolecule trapping one SO_4_^2−^ anion

The 2 M ZnSO_4_ aqueous solution with the merits of high ionic conductivity, low cost and adequate electrochemical stability window is widely applied for ZHSs [33–35] and thus is selected as the baseline electrolyte herein. As a low-cost and high-biosafety material in the field of medicine, the SC supramolecule used herein is synthesized by grafting two sulfobutyl groups to the external surface of β-cyclodextrin (β-CD, Fig. S1) which breaks the intramolecular hydrogen bonds in the β-CD and thus shows notably enhanced solubility of 150 mM in the ZnSO_4_ aqueous electrolyte (*vs.* 5 mM for the β-CD, Fig. S2). On this basis, the ZnSO_4_-based hybrid electrolytes with various concentrations of the SC additive are prepared (denoted as ZSO/SC-*x*, where *x* is the SC molar concentration) and they inherit the nonflammability merit of the ZnSO_4_ aqueous electrolytes (Fig. S3). Furthermore, as shown in Fig. 1a, with the increasing concentration of the SC additive, the pH value of the ZnSO_4_-SC hybrid electrolyte slightly decreases, whereas the viscosity rapidly increases and thus causes reduced ionic conductivity. Since low pH and poor ionic conductivity of electrolytes tend to aggravate dendrite growth and parasitic reactions of zinc anodes, high concentrations of the SC additive in the ZnSO_4_-based electrolytes are not preferred (which will be confirmed below by evaluating the electrochemical performance of zinc anodes in the ZnSO_4_-based hybrid electrolytes with various SC concentrations) [27, 29]. The solvation structure of the ZnSO_4_-SC hybrid electrolytes is analyzed through FTIR spectroscopy and Raman spectroscopy techniques. Compared with the pure ZnSO_4_ electrolyte, the ZnSO_4_-SC hybrid electrolytes present new adsorption bands on their FTIR spectra such as the C-O vibration at ~ 1042 cm^−1^ which originates from the SC supramolecules (Figs. 1b and S4), and the stretching vibration of the SO_4_^2−^ group appearing at 1075 cm^−1^ in the FTIR spectrum of the pure ZnSO_4_ electrolyte moves toward a higher wavenumber after the introduction of the SC additive, manifesting diminished constraint around the SO_4_^2−^, which implies that the introduced SC supramolecules weaken the Zn^2+^-SO_4_^2−^ coordination interaction [36–38]. Besides, the FTIR spectra in Fig. 1c show a continuous blue shift for the O–H bending vibration and O–H stretching vibration with increasing concentration of the SC additive in the ZnSO_4_-based electrolytes, demonstrating that the restrain enforced by the local environment on water molecules becomes weak and fewer water molecules adjoin each other to form strong H-bond [12, 39]. Quantitative analysis based on the Raman spectra of the ZnSO_4_-SC hybrid electrolytes in Figs. 1d and S5 provides a consistent conclusion that enhanced SC concentration in the hybrid electrolytes results in decreased proportion of the strong H-bond and increased proportions of the weak and medium H-bond. This indicates that the SC supramolecules break the continuous H-bond network in the bulk electrolytes, thereby limiting the Grotthuss mechanism-realized proton diffusion and will be helpful in inhibiting hydrogen evolution at the zinc deposition interface (Fig. S6) [40, 41].

MD simulations are further applied to reveal the solvation structure of the ZnSO_4_-SC hybrid electrolytes, as shown in Figs. 1e, f and S7. The primary solvation shell of Zn^2+^ contains 5.27 water molecules and 0.73 SO_4_^2−^ anions in the pure ZnSO_4_ electrolyte. The introduced SC supramolecules in the ZnSO_4_-SC hybrid electrolytes change the solvation structure of Zn^2+^, in which case Zn^2+^ coordinates with 0.16 SC supramolecules as well as 5.10 water molecules and 0.64 SO_4_^2−^ anions in the primary solvation shell. That is, the amount of water molecules and SO_4_^2−^ anions is reduced by the entry of the SC supramolecules in the primary solvation shell of Zn^2+^, which is beneficial for suppressing active water molecules and anions-involved parasitic reactions at the zinc deposition interface such as hydrogen evolution and corrosion [42].

DFT calculations help to understand the formation mechanisms of the aforementioned solvation structure of the ZnSO_4_-SC hybrid electrolytes (Fig. S8). Figure 1g summarizes the binding energy between different species in the ZnSO_4_-SC hybrid electrolytes. The mutual attraction between Zn^2+^ and water molecules corresponds to a binding energy of -0.25 eV, making water molecules coordinate with Zn^2+^ to form hydrated cations in aqueous zinc-salt electrolytes. The -OH and -SO_3_ groups in the SC supramolecules are electron-rich zones with negative electrostatic potentials (Fig. 1h), resulting in much stronger interactions with positively-charged Zn^2+^, whose binding energy reaches -1.14 and -1.63 eV, respectively. Therefore, the SC supramolecules are capable of entering the primary solvation shell of Zn^2+^ to prompt the release of solvated water molecules. Meanwhile, the interaction between water molecules and the functional groups in the SC supramolecules is stronger than that between water molecules (Fig. 1g), and each SC supramolecule contains a considerable amount of the functional groups including 19 -OH groups and 2 -SO_3_ groups, enabling the SC supramolecules to destroy the orderliness of water molecules arrangement in the bulk electrolytes. As a result, although the SC supramolecules cause the release of water molecules from the primary solvation shell of Zn^2+^, the continuity of the H-bond network in the ZnSO_4_-SC hybrid electrolytes is reduced as illustrated in Fig. 1c, d. Furthermore, the interior cavity of the SC supramolecule presents the capability of trapping SO_4_^2−^ anions (Fig. 1i). The DFT calculations show that each SC supramolecule trapping one SO_4_^2−^ anion is in the most thermodynamically stable state, in which case the system energy decreases by 2.63 eV. This feature of the SC supramolecules favors anion–cation decoupling to realize fast Zn^2+^ cation transport in the ZnSO_4_-SC hybrid electrolytes [32, 43]. To be specific, by calculating the mean-squared displacement of electrolyte ions over a period of time, the diffusion coefficient of Zn^2+^ cations increases from 1.03 × 10^–6^ cm^2^ s^−1^ for the ZnSO_4_ electrolyte to 1.25 × 10^–6^ cm^2^ s^−1^ for the ZSO/SC-10 electrolyte, while that of SO_4_^2−^ anions decreases by 19.5% before and after the introduction of the SC supramolecules in the ZnSO_4_-based electrolytes.

**Fig. 2:**
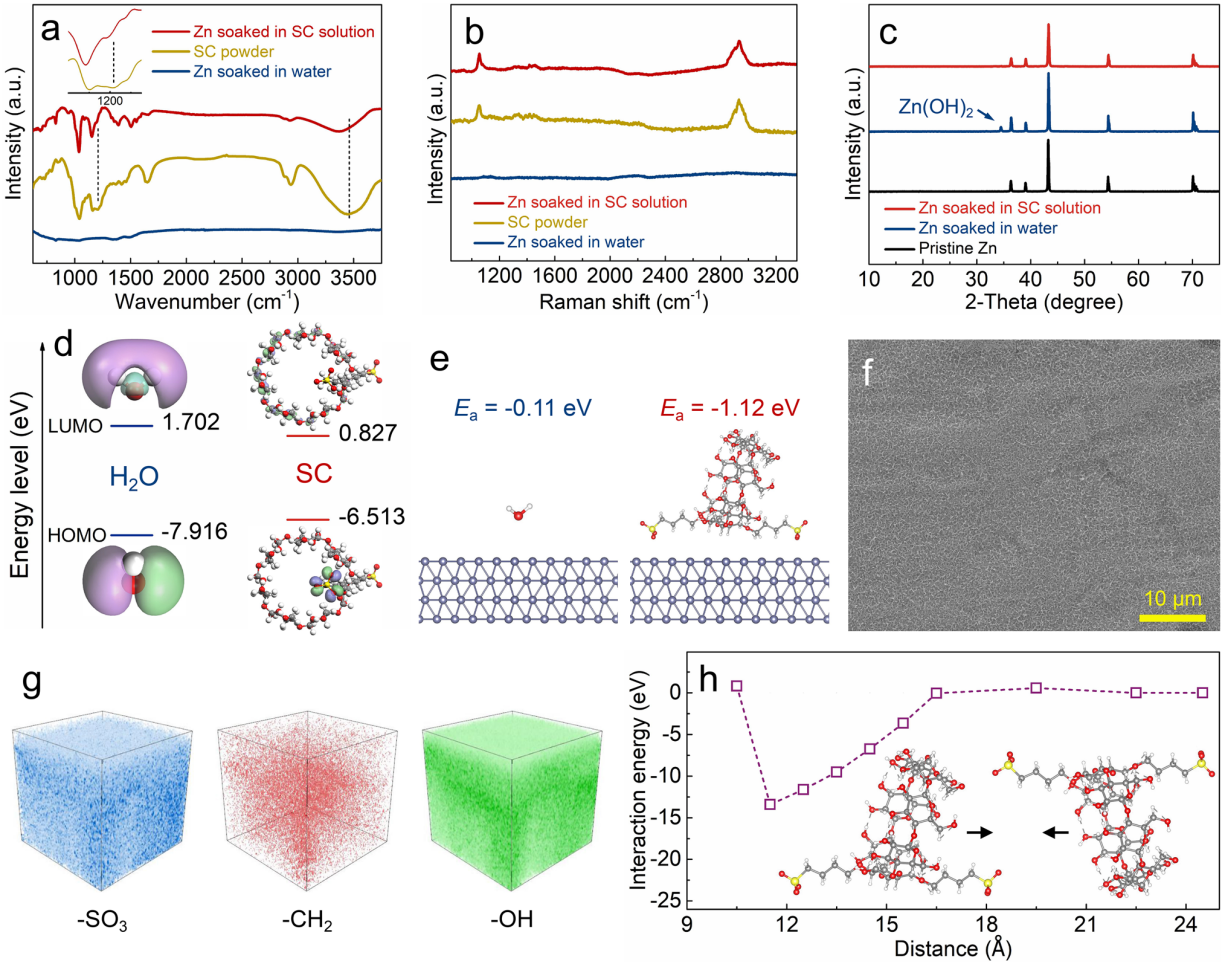
**a** FTIR spectra, **b** Raman spectra and **c** XRD patterns of zinc anodes after soaking in water and 10 mM SC aqueous solution. **d** Molecular orbital levels of the water molecule and the SC supramolecule. **e** Adsorption energy of water molecules (left) and SC supramolecules on zinc anodes. Micromorphology and component analysis of the SC supramolecules-assembled layer on zinc anodes: **f** SEM image and **g** 3D render for the representative elements of SC supramolecules obtained from TOF–SIMS. **h** The interaction energy between neighboring SC supramolecules at different distances

The SC supramolecule additive not only changes the solvation structure of the ZnSO_4_-based electrolytes, but also remodels the electrolyte–zinc anode interface. The ZnSO_4_-SC hybrid electrolytes display smaller contact angles and larger wetting free energies on the zinc anodes compared with the pure ZnSO_4_ electrolyte (Fig. S9), suggesting that the SC supramolecules have a high affinity to zinc anodes and thus strengthen the molecular interaction between the electrolytes and zinc anodes [44]. The affinity of the SC supramolecules to zinc anodes is intuitively substantiated by their spontaneous adsorption behavior on zinc anodes. As exhibited in Fig. 2a, b, after soaking zinc anodes into a 10 mM SC aqueous solution and then washing with water, the characteristic peaks of the SC supramolecules are detected from the FTIR and Raman spectra of the zinc anodes, and no other components appear, preliminarily demonstrating that the SC supramolecules adsorb while do not decompose on zinc anodes. For the SC supramolecules adsorbing on zinc anodes, the FTIR signal representing the -SO_3_ group at ~ 1205 cm^−1^ notably diminishes (inset in Fig. 2a) and other FTIR signals such as the O–H stretching vibration at around 3445 cm^−1^ also show varying shifts, which is because the electron-rich -SO_3_ group strongly interacts with the electron-poor cavity of the adjacent SC supramolecule as will be verified below. More analysis also proves the spontaneous adsorption of the SC supramolecules on zinc anodes (Fig. S10). According to DFT calculations in Fig. 2d, the SC supramolecule is characterized by a lower-energy lowest unoccupied molecule orbital (LUMO) and a higher-energy occupied molecule orbital (HOMO) than the water molecule. This means that the electron transfer between the SC supramolecule and metallic zinc is easier, thereby promoting the chemical adsorption of the SC supramolecules on zinc anodes [45]. As exhibited in Fig. 2e, the water molecule shows the tendency to adsorb on zinc anodes, with an adsorption energy of -0.11 eV, providing conditions of water-induced zinc anode corrosion, as evidenced by the generated corrosion product such as zinc hydroxide when soaking zinc anodes into pure water (Figs. 2c and S11) [46, 47]. In sharp contrast, the SC supramolecule stably adsorbs on the surface of metallic zinc, corresponding to an adsorption energy of -1.12 eV, which enables it superior to water to spontaneously adsorb on zinc anodes, and therefore, water-induced zinc anode corrosion is inhibited in the SC aqueous solution. In addition, theoretical calculations point out that trapping SO_4_^2−^ anions in the interior cavity slightly strengthens the spontaneous adsorption tendency of the SC supramolecules on zinc anodes (Fig. S12). The spontaneous adsorption of the SC supramolecules on zinc anodes is also verified when soaking zinc anodes in ZnSO_4_-SC hybrid electrolytes (Fig. S13). Moreover, the strong adsorption of the SC supramolecules induces their self-assembly into a dense layer on zinc anodes. SEM observation and time-of-flight secondary ion mass spectrometry (TOF–SIMS) analysis reveal the SC supramolecule-assembled dense structure on zinc anodes (Figs. 2f, g and S14). The interaction between the SC supramolecules is very weak in low-concentration aqueous solutions due to their large distance and the shielding effect of water molecules, whereas for the SC supramolecules adsorbing on zinc anodes, their extremely reduced distance results in significant intermolecular interaction. By calculating the interaction energy between adjacent SC supramolecules at different distances as shown in Figs. 2h and S15, it is revealed that as SC supramolecules approach each other, the mutual attraction between the electron-rich -SO_3_ group of one SC supramolecule and the electron-poor cavity of the adjacent SC supramolecule becomes stronger, in which case the system energy continuously decreases until the distance between adjacent SC supramolecules is smaller than 11.5 Å. Such mutual attraction between adjacent SC supramolecules results in the formation of an interconnecting state, which well explains the self-assembled dense structure of the SC layer on zinc anodes. This is also consistent with the FTIR analysis in Fig. 2a.

**Fig. 3:**
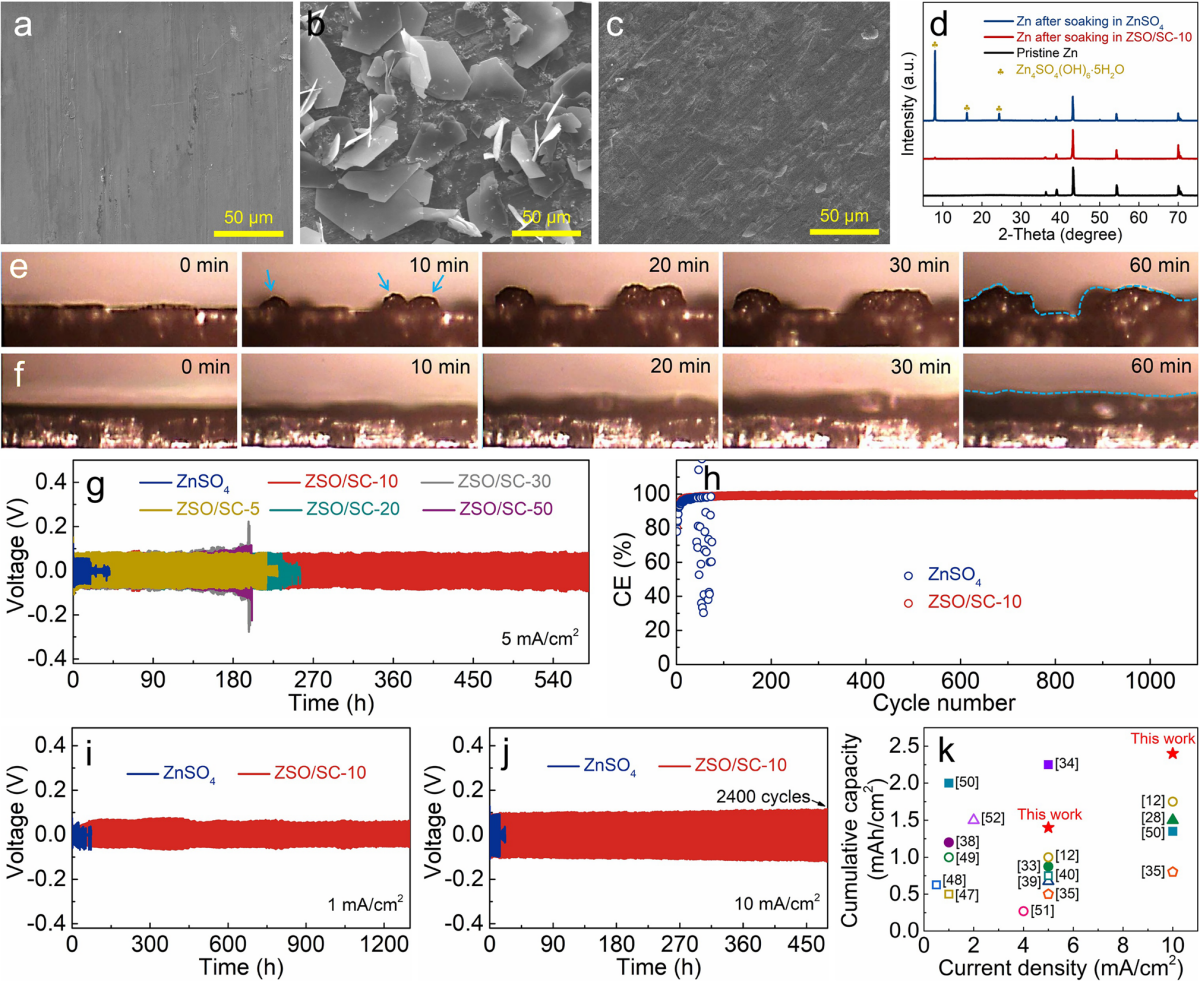
SEM images of zinc anodes **a** at initial state and after soaking in **b** ZnSO_4_ and **c** ZSO/SC-10 electrolytes for 2 days, and **d** corresponding XRD patterns. *In situ* optical images of the zinc deposition morphologies in **e** ZnSO_4_ and **f** ZSO/SC-10 electrolytes. **g** Cycling performance at 5 mA cm^−2^ and 2.5 mAh cm^−2^ of Zn//Zn symmetric cells using the ZnSO_4_-based electrolytes with various SC concentrations. **h** CE of Cu//Zn asymmetric cells using the ZnSO_4_ and ZSO/SC-10 electrolytes. Cycling stability of zinc anodes in the ZnSO_4_ and ZSO/SC-10 electrolytes at **i** 1 and **j** 10 mA cm^−2^ with a fixed capacity of 1 mAh cm^−2^. **k** Cumulative capacity of zinc anodes in various electrolytes as reported in the literature

The corrosion behavior of zinc anodes as well as zinc deposition morphology in the ZnSO_4_-based electrolytes is investigated first since these two factors significantly influence the electrochemical stability of zinc anodes. As exhibited in Fig. 3a-d, the soaking of zinc anodes in the pure ZnSO_4_ electrolyte causes the generation of a flake-like corrosion product, which is identified as basic zinc sulfate of Zn_4_SO_4_(OH)_6_·5H_2_O (PDF#78–0246) based on the XRD analysis. The generation of the basic zinc sulfate originates from the zinc metal corrosion-caused electrolyte pH increase (Fig. S16) and will destroy the electric field stability at the zinc deposition interface to induce zinc dendrite growth [8, 11]. Differently, the zinc anodes after soaking into the ZSO/SC-10 electrolyte keep flat surface morphology and there is almost no discernible corrosion product (Fig. 3c, d), proving that zinc anode corrosion is very weak in the ZSO/SC-10 electrolyte, which is necessary to maintain stable zinc deposition interface during the electrochemical reactions of zinc anodes. The XRD analysis also shows that there are no ZnS, ZnCO_3_ and other products which are possibly generated through the chemical reaction between zinc anodes and SC supramolecules, indicating the stable adsorption behavior of the SC supramolecules on zinc anodes. Furthermore, *in situ* optical observations are applied to study zinc deposition morphology in the ZnSO_4_-based electrolytes. As zinc deposition proceeds, small protuberance-like zinc deposits appear and then grow into large dendrites in the ZnSO_4_ electrolyte (Fig. 3e), while the deposited zinc in the ZSO/SC-10 electrolyte keeps a flat micromorphology without the formation of notable protuberances (Fig. 3f). This testifies that the SC supramolecules are capable of inducing dendrite-free zinc deposition. *In situ* Raman analysis in Fig. S17 also verifies that the SC supramolecules stabilize the zinc deposition interface.

The electrochemical stability of zinc anodes in various ZnSO_4_-based electrolytes is evaluated. As shown in Fig. 3g, the Zn//Zn symmetric cells using the ZnSO_4_ electrolyte suffer from short circuit after operating for only 19 h under the testing conditions of 5 mA cm^−2^ and 2.5 mAh cm^−2^. The application of the ZnSO_4_-SC hybrid electrolytes remarkably extends the operation lifetime of the Zn//Zn symmetric cells. Especially, the operation lifetime of the symmetric cells utilizing the ZSO/SC-10 electrolyte exceeds 580 h, 30 times longer than that of the symmetric cells with the ZnSO_4_ electrolyte. This can be ascribed to the repressed corrosion reactions and dendrite growth on zinc anodes by the ZSO/SC-10 electrolyte (Fig. S18). Note that a relatively high SC concentration such as 50 mM hampers the Zn^2+^ transport and desolvation process at the zinc deposition interface which are detrimental to dendrite-free zinc deposition, thereby causing the modest electrochemical stability of zinc anodes. Therefore, the reversibility and electrochemical stability of zinc anodes in the ZnSO_4_-based hybrid electrolyte with 10 mM SC concentration are further investigated. Zinc plating/stripping under the testing conditions of 2 mA cm^−2^ and 1 mAh cm^−2^ in the ZSO/SC-10 electrolyte is very stable over 1100 cycles, and a superior average Coulombic efficiency (CE) of 99.3% is achieved (Figs. 3h and S19). In sharp contrast, stable zinc plating/stripping in the pure ZnSO_4_ electrolyte is only realized in the initial 45 cycles with an average CE of 94.2%, followed by abnormal zinc plating/stripping behaviors and dramatically fluctuating CE values. A similar phenomenon is observed when the zinc plating/stripping process is performed at an enhanced current density such as 5 mA cm^−2^ that the ZSO/SC-10 electrolyte helps to realize an average CE of 99.7% over 770 cycles (Fig. S20). These manifest that the reversibility of zinc anodes is markedly enhanced in the ZSO/SC-10 electrolyte. As expected, zinc anodes in the ZSO/SC-10 electrolyte exhibit superior electrochemical stability at a wide range of operating current densities and zinc plating capacities (Figs. 3i, j and S21, S22). It is worth noting that even at large current densities such as 10 mA cm^−2^, zinc anodes in the ZSO/SC-10 electrolyte stably work for at least 2400 cycles (*i.e.*, 480 h), which is 34 times the cycle number for the zinc anodes in the pure ZnSO_4_ electrolyte. Correspondingly, the cumulative capacity of zinc anodes working in the ZSO/SC-10 electrolyte exceeds 2.4 Ah cm^−2^, distinctly better than the performance achieved in many other electrolytes as summarized in Fig. 3k and Table S1 [12, 27, 32–34, 37–39, 46–51], not only reflecting the high structure robustness of the interconnecting SC supramolecule interface in durably stabilizing zinc anodes but also enabling the ZSO/SC-10 electrolyte to be used for ultralong-life and high-power zinc-based electrochemical energy storage such as ZHSs.

**Fig. 4:**
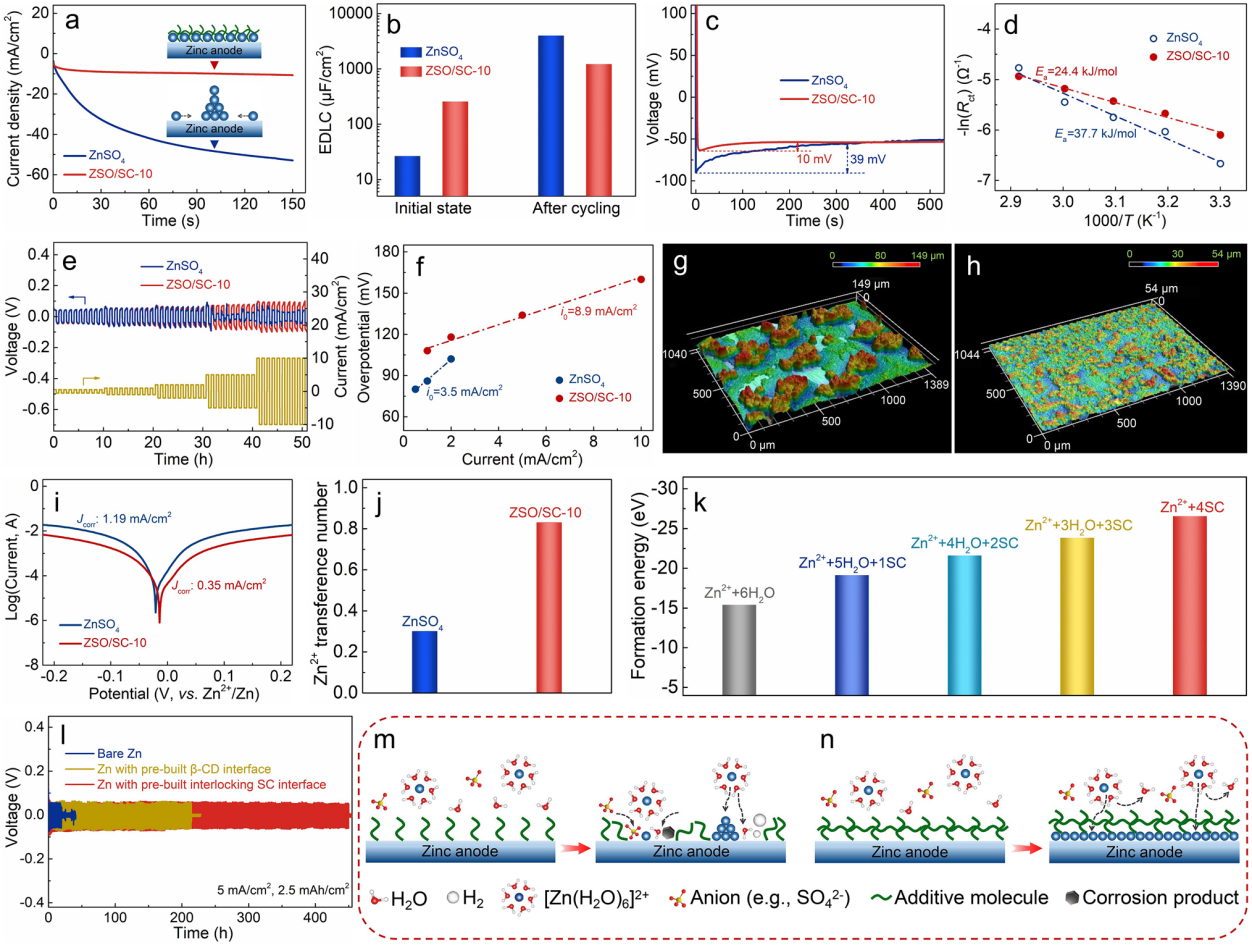
Zinc deposition behavior study in the ZnSO_4_ and ZSO/SC-10 electrolytes: **a** CA curves of the Zn//Zn symmetric cells under an overpotential of -150 mV; **b** EDLC of the zinc anode/electrolyte interface in the Zn//Zn symmetric cells before and after cycling; **c** nucleation overpotential curves for the zinc deposition at 2 mA cm^−2^; **d** activation energy of Zn^2+^ desolvation; **e** rate performance of the Zn//Zn symmetric cells and **f** corresponding exchange current density at the zinc deposition interface; LSCM images of electrodeposited zinc in **g** the ZnSO_4_ and **h** ZSO/SC-10 electrolytes (electrodeposition parameters: 2 mA cm^−2^ and 10 mAh cm^−2^); **i** tafel plots of the Zn//Zn symmetric cells;** j** Zn^2+^ transference number; **k** formation energy of Zn^2+^ solvation structures containing varying numbers of water and SC supramolecules; **l** cycling stability in pure ZnSO_4_ electrolyte of zinc anodes with pre-built molecule interfaces; schematics illustrating zinc deposition behaviors at different molecule interfaces on zinc anodes: **m** discrete molecule interface and **n** interconnecting molecule interface

To understand the regulation mechanisms of the SC supramolecule additive on zinc anodes, the zinc deposition behaviors in the ZnSO_4_ and the ZSO/SC-10 electrolytes are deeply analyzed. As shown in Fig. 4a, the CA curve of Zn//Zn symmetric cells using the ZnSO_4_ electrolyte exhibits continuously increasing response current, suggesting an uncontrolled 2D diffusion of Zn^2+^ at the zinc deposition interface to trigger zinc dendrite growth [23, 52]. The response current in the case of the ZSO/SC-10 electrolyte increases only within the initial several seconds and then remains constant throughout the whole zinc deposition process, which demonstrates that the lateral diffusion of Zn^2+^ and its triggered dendrite growth at the zinc deposition interface are restrained. This benefits from the highly zincophilic –OH and –SO_3_ functional groups of the SC supramolecules (as have been discussed in Fig. 1g), which serve as abundant Zn^2+^ adsorption sites in the interconnecting SC supramolecule interface to impede the rampant 2D diffusion of Zn^2+^ at the zinc deposition interface [53]. The EDLC of the electrolyte–zinc anode interface is 257 μF cm^−2^ in the ZSO/SC-10 electrolyte (Figs. 4b and S23), much larger than that achieved in the pure ZnSO_4_ electrolyte (27 μF cm^−2^), also demonstrating the abundant Zn^2+^ adsorption sites at the zinc deposition interface in the SC-containing hybrid electrolyte [23]. Note that the EDLC of the electrolyte–zinc anode interface dramatically increases by 150 times after repeatedly zinc plating/stripping in the ZnSO_4_ electrolyte, which is due to the formation of large-specific-surface-area zinc dendrites, whereas the increased amplitude of the EDLC value is much smaller in the ZSO/SC-10 electrolyte, indicating the inhibition of dendrite growth at the interconnecting SC supramolecule–zinc anode interface. In addition, the abundant zincophilic sites at the interconnecting SC supramolecule interface on zinc anodes are conducive to reducing the nucleation barrier during the zinc deposition process [54]. Specifically, the nucleation overpotential during zinc deposition at 2 mA cm^−2^ decreases from 39 mV in the ZnSO_4_ electrolyte to 10 mV in the ZSO/SC-10 electrolyte (Fig. 4c). Zinc deposition behaviors at large current densities also confirm this (Fig. S24). Meanwhile, since the desolvation is the rate-limiting step of the zinc deposition process [5, 6], the activation energy of the Zn^2+^ desolvation process is determined based on the Arrhenius equation in Figs. 4d and S25, which is 24.4 kJ mol^−1^ in the ZSO/SC-10 electrolyte, notably lower than that in the ZnSO_4_ electrolyte (37.7 kJ mol^−1^). Benefiting from the easier desolvation–nucleation process in the ZSO/SC-10 electrolyte, fast zinc plating/stripping is realized for the zinc anodes. As exhibited in Fig. 4e, with the current density increasing from 0.5 to 10 mA cm^−2^ at a fixed charge/discharge cycle time of 1 h, the Zn//Zn symmetric cell using the ZSO/SC-10 electrolyte always presents normal charge/discharge behaviors, while that using the ZnSO_4_ electrolyte is crippled when the current density reaches 5 mA cm^−2^. Accordingly, the exchange current density of the zinc plating/stripping reaction in the symmetric cell using the ZSO/SC-10 and the ZnSO_4_ electrolytes is calculated to be 8.9 and 3.5 mA cm^−2^, respectively (Fig. 4f). These reflect that the Zn^2+^ reduction at the zinc deposition interface is easier in the ZSO/SC-10 electrolyte, which helps to retard uncontrollable Zn^2+^ aggregation-triggered dendrite growth. Therefore, zinc deposition in the ZSO/SC-10 electrolyte is invested with abundant low-barrier nucleation sites, fast desolvation kinetics and easy reduction feature, thereby achieving dendrite-free and dense morphology. As examined by the 3D LSCM images in Fig. 4g, h, zinc deposition in the ZnSO_4_ electrolyte tends to form sparse large-sized zinc dendrites whose height mostly exceeds about 120 μm, whereas electrodeposited zinc in the ZSO/SC-10 electrolyte exhibits the morphology of uniformly dispersed small-sized particles with close heights (*e.g.*, the average surface roughness is only 5.6 μm). Consistently, zinc deposition in the ZSO/SC-10 electrolyte tends to form a dense morphology at various current densities, which is significantly different from the incompact morphology of electrodeposited zinc in the ZnSO_4_ electrolyte (Fig. S26).

The superior electrochemical performance of zinc anodes in the ZSO/SC-10 electrolyte is also associated with suppressed parasitic reactions at the interconnecting SC supramolecule–zinc anode interface. According to Tafel plots of Zn//Zn symmetric cells in Fig. 4i, the corrosion potential for zinc anodes improves from -21 to -14 mV and the corrosion current reduces from 1.2 to 0.4 mA cm^−2^ after the introduction of the SC supramolecules in ZnSO_4_-based electrolytes, indicating an enhanced anti-corrosion ability [55]. Moreover, since the strong solvation effect restricts Zn^2+^ transport in ZnSO_4_ electrolytes [26, 56], the Zn^2+^ transference number is only 0.30 in the ZnSO_4_ electrolyte (Figs. 4j and S27), making SO_4_^2−^ anions easy to enter the zinc deposition interface to corrode zinc anodes and magnifying concentration polarization at the zinc deposition interface to promote zinc dendrite growth [52, 53]. Inspiringly, a very high Zn^2+^ transference number of 0.83 is achieved when the ZSO/SC-10 electrolyte is applied. This is because the interior cavity of the SC supramolecules is capable of trapping SO_4_^2−^ anions (as discussed in Fig. 1i), and therefore, the interconnecting SC supramolecule interface on zinc anodes serves as an effective barrier retarding SO_4_^2−^ passage. As a result, the issues of concentration polarization-promoted zinc dendrite growth and anions-involved corrosion will be alleviated at the zinc deposition interface. In addition, according to the DFT calculations in Figs. 4k and S28, when hydrated Zn^2+^ from the electrolyte passes through the interconnecting SC supramolecule interface to deposit on zinc anodes, the removal of the water molecules from the primary solvation shell of Zn^2+^ is thermodynamically favorable since the solvated Zn^2+^ structure with more SC supramolecules (*i.e.*, less water molecules) corresponds to lower energy, thus improving the desolvation kinetics of hydrated Zn^2+^ and reducing the active water molecules that can induce anode corrosion and hydrogen evolution reactions at the zinc deposition interface. The above analysis illustrates the vital role of the interconnecting SC supramolecule interface in positively regulating zinc deposition behaviors. As proof, pre-built interconnecting SC supramolecule interface-modified zinc anodes which are designed by soaking zinc anodes in 10 mM SC aqueous solution present an outstanding operation lifetime of 450 h (*vs.* 19 h for bare zinc anodes) at 5 mA cm^−2^ and 2.5 mAh cm^−2^ (Fig. 4l). It is worth emphasizing that such a performance is slightly inferior to that of the zinc anodes in the ZSO/SC-10 electrolyte because the SC supramolecules in the ZSO/SC-10 hybrid electrolyte not only built an interlocking SC interface on zinc anodes but also remodel the electrolyte structure (*e.g.*, optimizing zinc-ion solvation structure and breaking the continuous H-bond network in the bulk electrolyte to inhibit parasitic reactions at the zinc deposition interface). Furthermore, if removing the sulfobutyl groups from the SC supramolecules, the obtained β-CD supramolecule can also adsorb on zinc anodes to optimize zinc plating/stripping behaviors [32, 34], while the absence of -SO_3_ group branches on its exterior of the cavity ring decides that adjacent β-CD supramolecules will not connect to form a robust interconnecting molecule interface, and therefore, pre-built β-CD interface-modified zinc anodes show a restricted electrochemical stability (Figs. 4l and S29). These emphasize the superiority of the interconnecting molecule interface in achieving long-term stable zinc anodes, as illustrated in Figs. 4m, n.

**Fig. 5:**
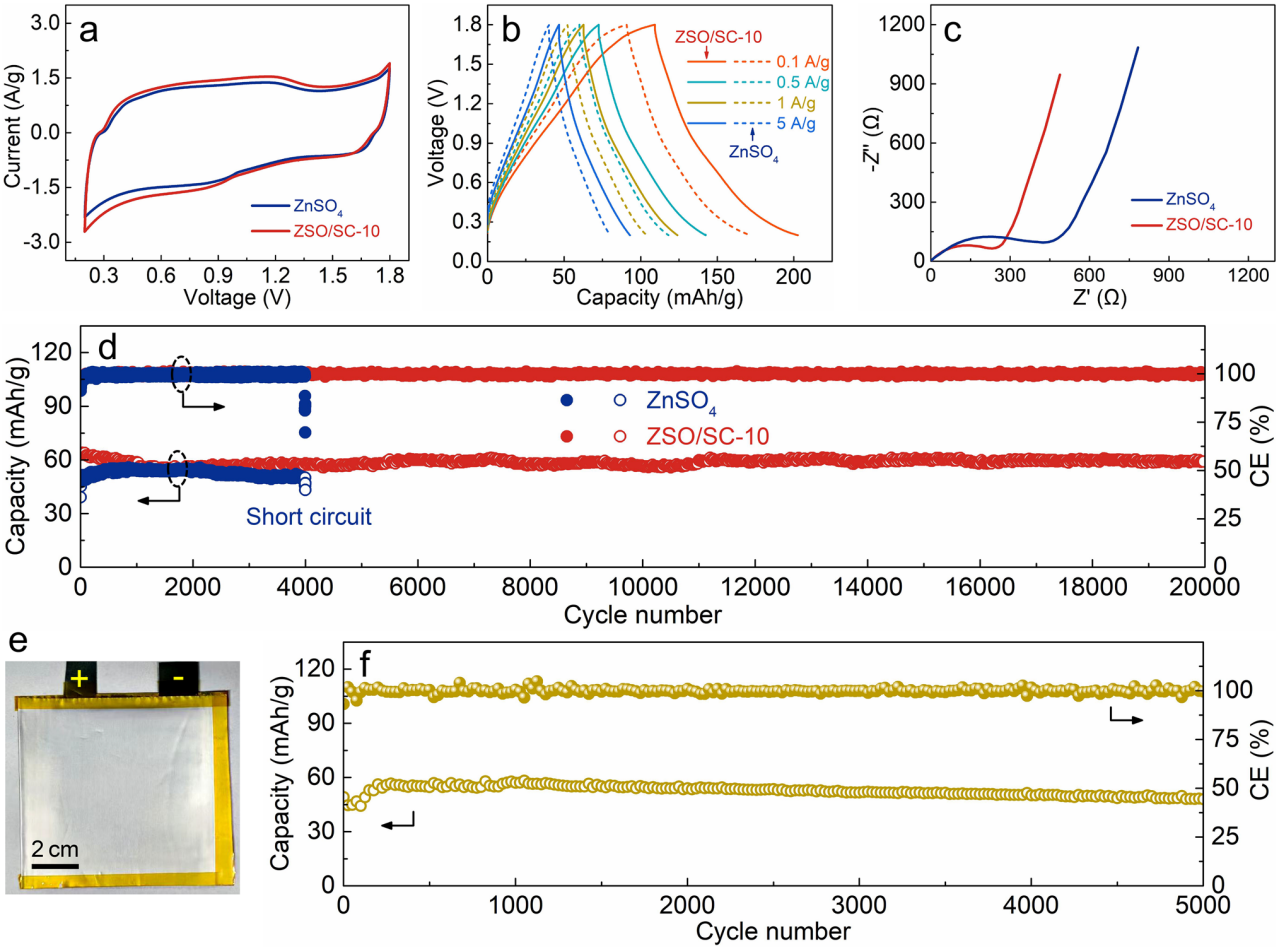
Electrochemical performance of AC//Zn ZHSs with the ZnSO_4_ and ZSO/SC-10 electrolytes: **a** CV curves at 10 mV s^−1^; **b** GCD profiles at 0.1–5 A g^−1^; **c** EIS spectra; **d** cycling performance at 1 A g^−1^. **e** Digital photo and **f** cycling performance at 1 A g^−1^ of pouch cell-typed AC//Zn ZHSs using the ZSO/SC-10 electrolyte

Benefiting from the optimizing effect on the electrochemical stability and kinetics of zinc anodes, the ZSO/SC-10 electrolyte is promising to be used for ultralong-life and high-power zinc-based electrochemical energy storage. As a demonstration, Zn//activated carbon (AC, Fig. S30) hybrid supercapacitors (*i.e.*, ZHSs) with the ZSO/SC-10 electrolyte were assembled. Compared with the Zn//AC ZHSs using the ZnSO_4_ electrolyte, the ZSO/SC-10 electrolyte-based ZHSs possess similar CV curves (Fig. 5a), demonstrating that the SC supramolecule additive in the ZSO/SC-10 electrolyte does not change the basic electrochemical energy storage mechanism of the ZHSs, *i.e.*, zinc plating/stripping at the zinc anodes and electrolyte ion adsorption/desorption at the carbon cathodes [1]. Considering the trace amount of the SC supramolecules in the ZSO/SC-10 electrolyte, the concentration reduction of SO_4_^2−^ anions caused by SC supramolecule trapping is minimal, and meanwhile, the SC supramolecules are capable of inhibiting anions-participated corrosion reactions at the zinc anode interface to stabilize the SO_4_^2−^ anion concentration of the electrolyte; thus, the anion-participated charge storage process of the AC cathode is not negatively affected. Based on the GCD and EIS tests in Fig. 5b, c, the ZHSs using the ZSO/SC-10 electrolyte present both higher capacity, superior rate performance and smaller charge transfer resistance than those using the ZnSO_4_ electrolyte, which is contributed by the enhanced electrochemical kinetics of zinc anodes in the ZSO/SC-10 electrolyte. Moreover, the ZHSs using the ZnSO_4_ electrolyte fail after only ~ 4000 charge/discharge cycles, ending by short circuit and low CE values, as displayed in Fig. 5d. On the contrary, since the ZSO/SC-10 electrolyte is capable of remarkably optimizing the electrochemical stability and reversibility of zinc anodes, its application enables the ZHSs to stably operate over 20,000 charge/discharge cycles, accompanied with a high capacity retention of 95% and almost constant CE of 100%. Even when the ZSO/SC-10 electrolyte is employed for pouch cell-typed AC//Zn ZHSs, in which the mass loading of the AC active material reaches 23.6 mg and the area of the zinc anode exceeds 20.0 cm^2^, respectable capacity and cycling property are realized (Fig. 5e, f). Due to the large size and high mass loading of the AC cathode in the pouch cell-typed ZHSs (*vs.* coin cell-typed ZHSs), the electrolyte needs a longer time to infiltrate the cathode, thereby resulting in a capacity increase of the pouch cell-typed ZHSs during the initial charge/discharge cycles. Besides ZHSs, the ZSO/SC-10 electrolyte presents notable superiority to the ZnSO_4_ electrolyte in the aspects of achieving high capacity, outstanding rate performance and good cycling stability of the Zn//VO_2_ ZIBs (Figs. S30 and S31). These verify the reliability of the ZSO/SC-10 electrolyte for optimizing the electrochemical stability and kinetics of zinc anodes in various zinc-based electrochemical energy storage systems.

## Conclusions

In summary, an electrolyte additive-assembled interconnecting molecules–zinc anode interface was proposed to realize highly stable and fast-kinetics zinc anodes for ZHSs. The ZnSO_4_-SC hybrid electrolytes not only inherited the nonflammability merit of the ZnSO_4_ aqueous electrolytes but also maintained high ionic conductivity and low viscosity at appropriate SC concentrations. The SC supramolecules reduced the coordination number of water molecules and SO_4_^2−^ anions with Zn^2+^ by entering into the primary solvation shell of Zn^2+^ and attracted water molecules to break continuous H-bond network, which helped to inhibit hydrogen evolution at the zinc deposition interface. More importantly, the SC supramolecules spontaneously adsorbed on zinc anodes due to their high affinity with metallic zinc and then self-assembled into an interconnecting molecules–zinc anode interface through the mutual attraction between the electron-rich sulfobutyl group and the electron-poor cavity of the adjacent SC supramolecule. Experimental analysis and theoretical computations pointed out that the interconnecting SC molecules–zinc anode interface provided abundant anion-trapping cavities and electron-rich zincophilic groups to enhance Zn^2+^ transference number and homogenize Zn^2+^ deposition sites, and meanwhile, it accelerated the desolvation of hydrated Zn^2+^ to promote zinc deposition kinetics and inhibit active water molecules from inducing parasitic reactions at the zinc deposition interface. As a consequence, the interconnecting SC molecules–zinc anode interface endowed zinc anodes with superior reversibility, significantly extended lifetimes and optimized rate performance, making it possible to realize ultralong-life and high-rate ZHSs. This work not only proposes a scalable strategy to achieve highly stable and fast-kinetics zinc anodes for ZHSs, but also provides new inspiration for the elaborate design of molecule–metal anode interfaces toward high-performance metal anode-based electrochemical energy storage.

## Supplementary Information

Below is the link to the electronic supplementary material.

Supplementary file1 (DOCX 44637 kb)
