# Supplementary material for: Electrolyte Additive-Assembled Interconnecting Molecules–Zinc Anode Interface for Zinc-Ion Hybrid Supercapacitors
Source: Nanomicro Lett. 2025 May 21;17:268. doi: 10.1007/s40820-025-01794-1 (PMC12095121; doi:10.1007/s40820-025-01794-1)
Supplement: Supplementary file 1 — Supplementary file1 (DOCX 44637 kb) [file 40820_2025_1794_MOESM1_ESM.docx]

Supporting Information for

**Electrolyte Additive-Assembled Interconnecting Molecules-Zinc Anode Interface for Zinc-Ion Hybrid Supercapacitors**

Yang Li ^1,2^, Xu Li ^3^, Xinya Peng ^3^, Xinyu Yang ^3^, Feiyu Kang ^2,^*, Liubing Dong ^3,^*

^1^ School of Materials and Energy, Foshan University, Foshan 528000, P. R. China

^2^ Tsinghua Shenzhen International Graduate School, Tsinghua University, Shenzhen 518055, P. R. China

^3^ College of Chemistry and Materials Science, Jinan University, Guangzhou 511443, P. R. China

*Corresponding authors. E-mail: [fykang@sz.tsinghua.edu.cn](mailto:fykang@sz.tsinghua.edu.cn) (Feiyu Kang ); [donglb@jnu.edu.cn](mailto:donglb@jnu.edu.cn) (Liubing Dong )

**Supplementary Figures and Table**


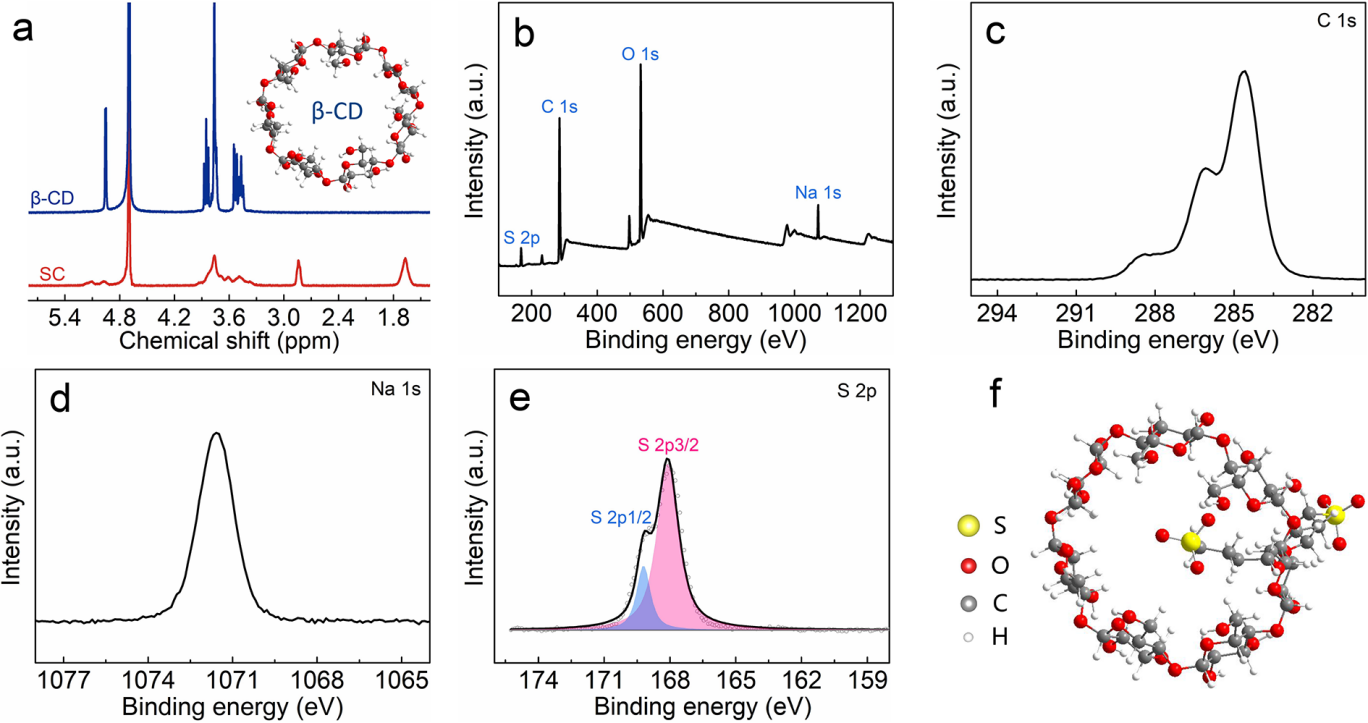


**Fig. S1** **a** ^1^H NMR spectra of the β-cyclodextrin (β-CD) and SC powders (inset: chemical structure of the β-CD). XPS analysis of the SC powder: **b** XPS full spectrum and fine spectra of **c** C 1s, **d** Na 1s and **e** S 2p. **f** Chemical structure of the SC. Compared with the ^1^H NMR spectrum of the β-CD, the newly appeared chemical shift peaks at 1.7 and 2.7 ppm for the SC sample suggest that some H atoms in the β-CD are replaced by sulfobutyl groups (-CH_2_-CH_2_-CH_2_-CH_2_-SO_3_) in the SC. For the XPS fine spectrum of S 2p in **e**, the peak positions of the S 2p1/2 and S 2p3/2 are consistent with those of sulfonate reported in the literature [S1], confirming the existence of -SO_3_ group in the SC powder. According to the atomic content of the C, Na and S elements, it is determined that there are about two sulfobutyl groups on average in each SC supramolecule. Based on the ^1^H NMR and XPS analysis, as well as DFT calculations, the thermodynamically favorable chemical structure of the SC is illustrated in **f**.


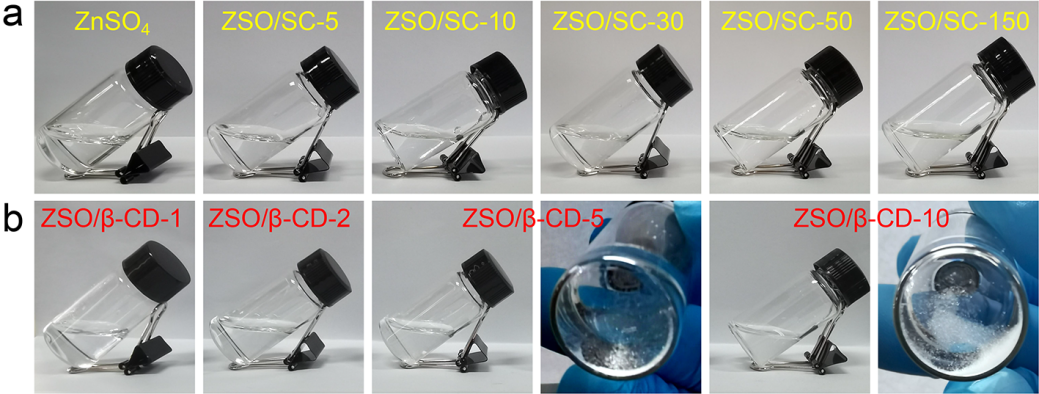


**Fig. S2** The solubility of **a** SC powder and **b** β-cyclodextrin (β-CD) powder in 2 M ZnSO_4_ electrolyte. “ZSO/β-CD-*y*” (*y*=1, 2, 5 and 10) in **b** means 2 M ZnSO_4_ electrolyte with *y* mM β-CD additive. The SC powder shows high solubility in 2 M ZnSO_4_ electrolytes, and therefore homogenous ZnSO_4_-SC hybrid electrolytes with SC concentration of 0-150 mM can be obtained. Note that this work focuses on the ZnSO_4_-SC hybrid electrolytes with the SC concentration of 0-50 mM because when the SC concentration exceeds 50 mM, the viscosity of the ZnSO_4_-SC hybrid electrolytes is too high to cause poor electrochemical performance for zinc anodes, and meanwhile, a high SC concentration means increased cost for the ZnSO_4_-based electrolytes. In contrast, the β-CD powder becomes insoluble when its concentration in 2 M ZnSO_4_ electrolyte reaches 5 mM.


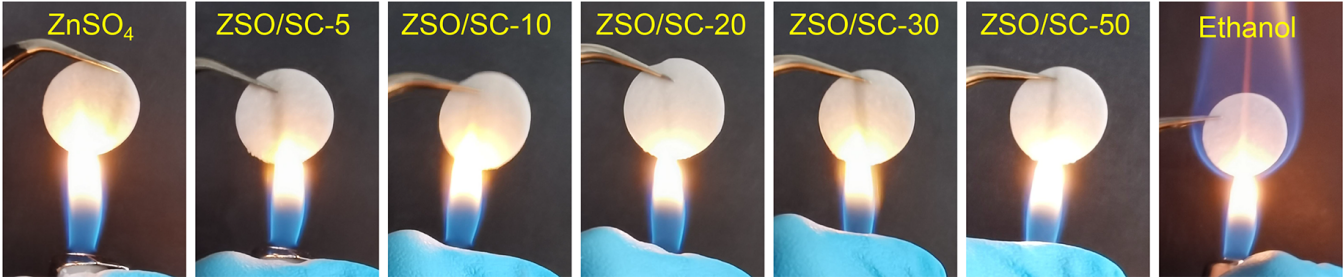


**Fig. S3** Flammability tests of the various solutions-saturated glass fiber membranes. For an intuitive understanding, a corresponding image of flammable ethanol is also displayed (the right-most image). The aqueous ZnSO_4_-SC hybrid electrolytes are nonflammable.


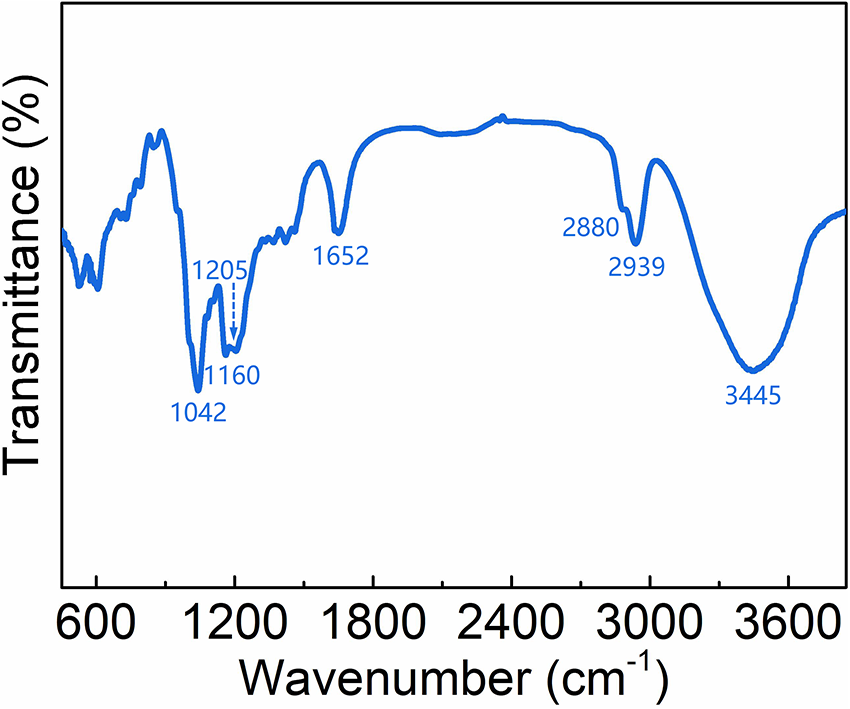


**Fig. S4** FTIR spectrum of the SC powder. The adsorption bands at 3445 and 1652 cm^-1^ correspond to the O-H stretching vibration and the O-H bending vibration in the hydroxyl groups, respectively. The C-H stretching vibrations in the carbon skeleton appear at 2939 and 2880 cm^-1^, and the C-O vibrations appear at 1160 and 1042 cm^-1^. Moreover, the adsorption band at 1205 cm^-1^ is attributed to the asymmetric stretching vibration of the S=O in the sulfonic acid group [S2].


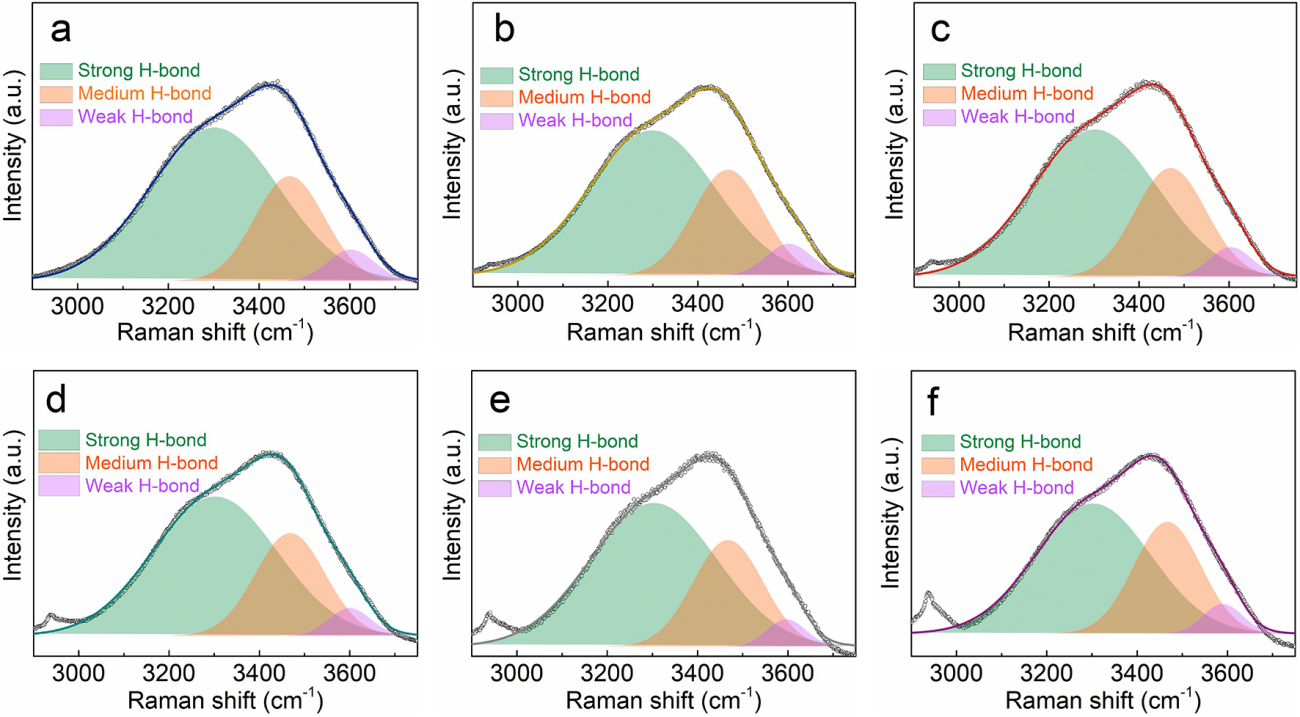


**Fig. S5** Raman spectra of the different electrolytes: **a** the pure ZnSO_4_, **b** ZSO/SC-5, **c** ZSO/SC-10, **d** ZSO/SC-20, **e** ZSO/SC-30 and **f** ZSO/SC-50. The Raman band at 2936 cm^-1^ originates from the C-H stretching vibration in the SC supramolecules.





**Fig. S6** LSV curves of Zn//Ti asymmetric cells using the ZnSO_4_ and ZSO/SC-10 electrolytes. The ZSO/SC-10 electrolyte displays a lower hydrogen evolution potential and a higher oxygen evolution potential, which illustrates that the SC additive in aqueous solutions is capable of inhibiting water activity. Meanwhile, the SC supramolecule additive is electrochemically stable in the operating voltage window, which suggests that the SC supramolecule additive will not decompose or react with other components in the electrolyte.

**
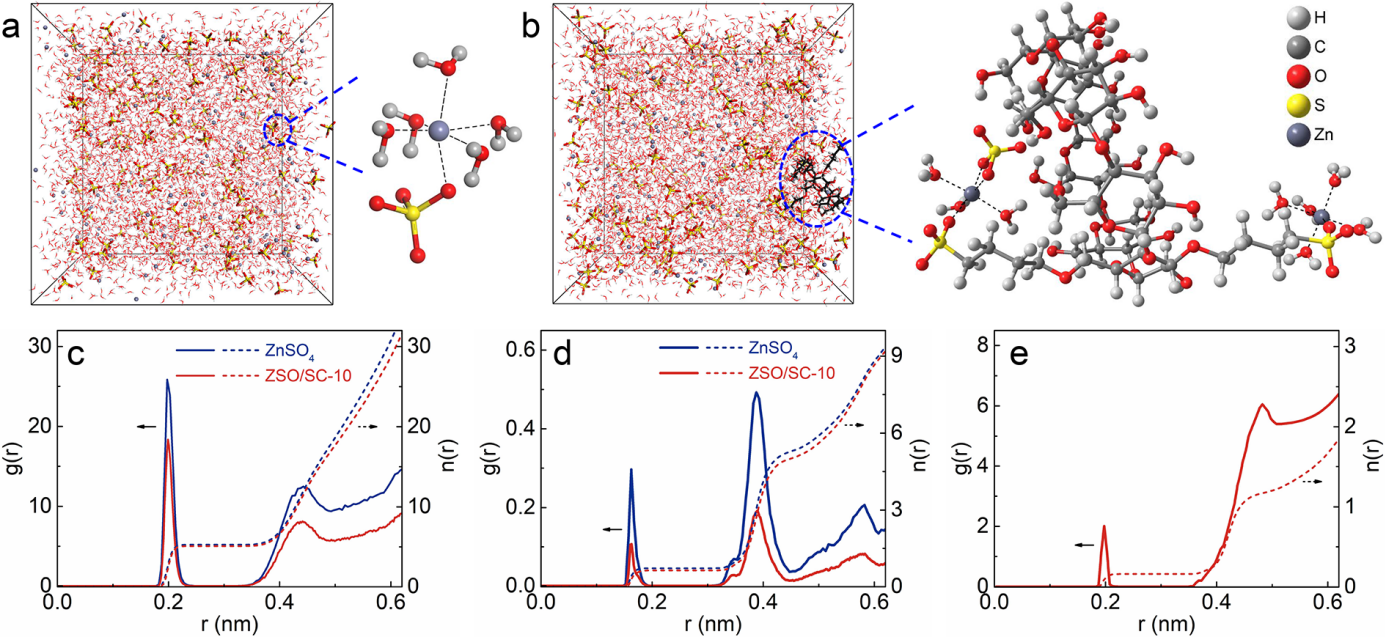
**

**Fig. S7** MD simulation results: 3D snapshot and enlarged primary solvation shell of Zn^2+^ in **a** the ZnSO_4_ and **b** ZSO/SC-10 electrolytes. Radial distribution functions (RDFs) for **c** Zn-O(H_2_O), **d** Zn-O(SO_4_^2-^) and **e** Zn-O(SC) in the above two electrolytes

**
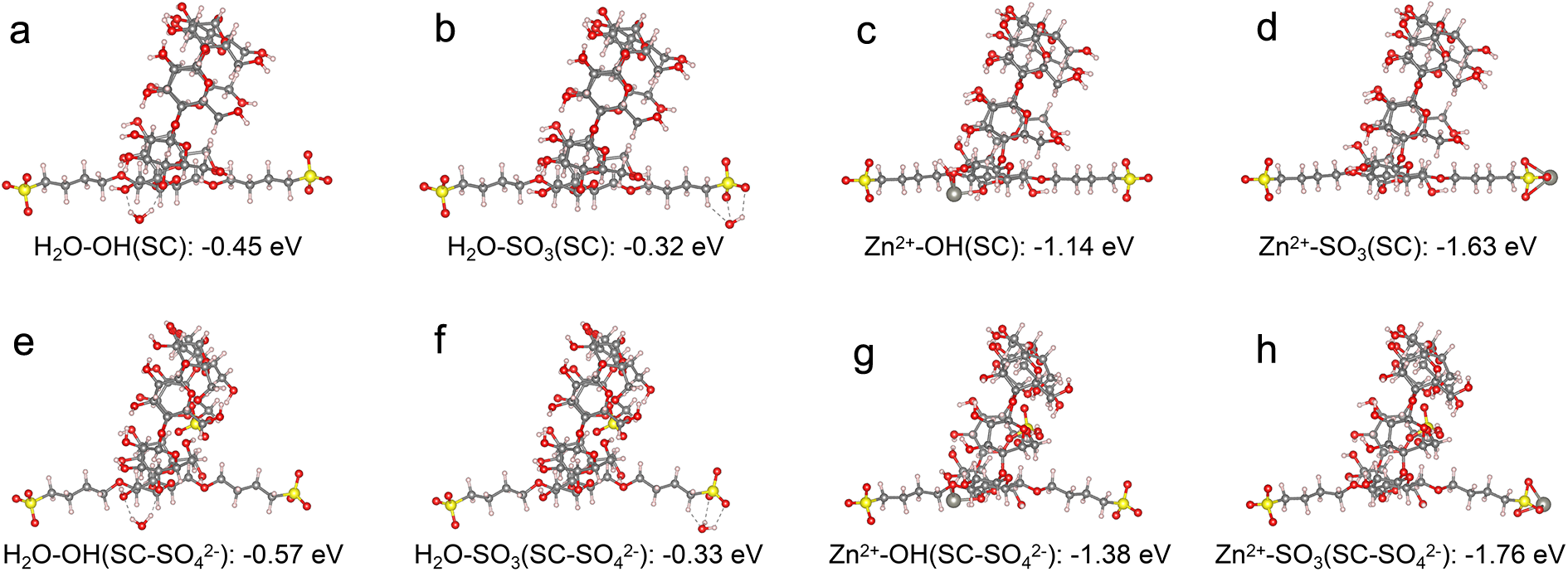
**

**Fig. S8** Thermodynamically optimal models of the interaction between the H_2_O molecule (or Zn^2+^) and the functional groups (*e.g.*, -OH and -SO_3_) of the SC supramolecule: **a** H_2_O-OH(SC); **b** H_2_O-SO_3_(SC); **c** Zn^2+^-OH(SC); **d** Zn^2+^-SO_3_(SC). Thermodynamically optimal models of the interaction between H_2_O molecule (or Zn^2+^) and the functional groups of the SC supramolecule after trapping one SO_4_^2-^ anion in the cavity: **e** H_2_O-OH(SC-SO_4_^2-^); **f** H_2_O-SO_3_(SC-SO_4_^2-^); **g** Zn^2+^-OH(SC-SO_4_^2-^); **h** Zn^2+^-SO_3_(SC). The H_2_O-SC supramolecule interaction and the Zn^2+^-SC supramolecule interaction are not weakened after the SC supramolecule traps one SO_4_^2-^ anion in the interior cavity. The violet, red, dark-grey, pale pink and yellow balls represent Zn, O, C, H and S atoms, respectively.


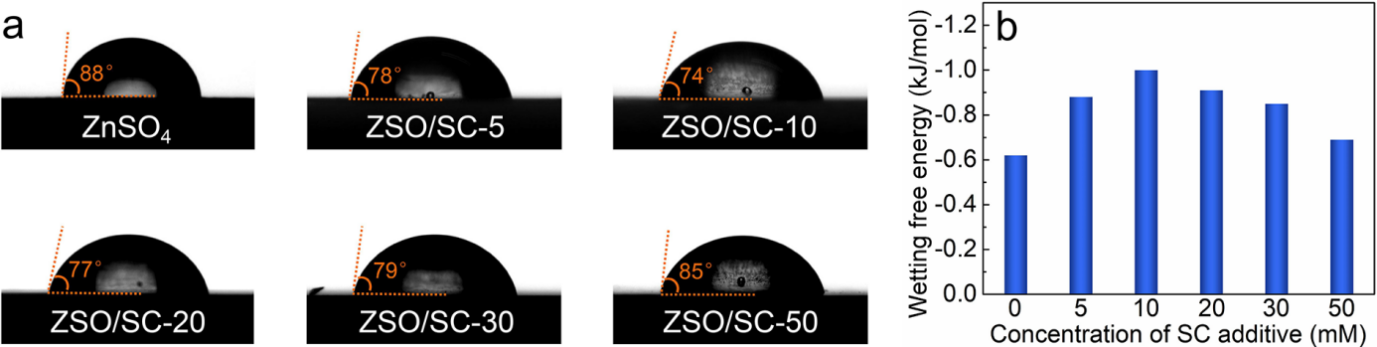


**Fig. S9** **a** Contact angles and **b** wetting free energy of various electrolytes on zinc anodes. The ZnSO_4_-SC hybrid electrolytes possess smaller contact angles and larger wetting free energies on the zinc anodes compared with the pure ZnSO_4_ electrolyte. Especially, the ZSO/SC-10 electrolyte shows the smallest contact angle of 74° as well as the maximum wetting free energy of -1.00 kJ/mol. For the hybrid electrolytes with relatively high SC concentrations such as the ZSO/SC-50 electrolyte, the large viscosity hinders their wetting on zinc anodes.

**
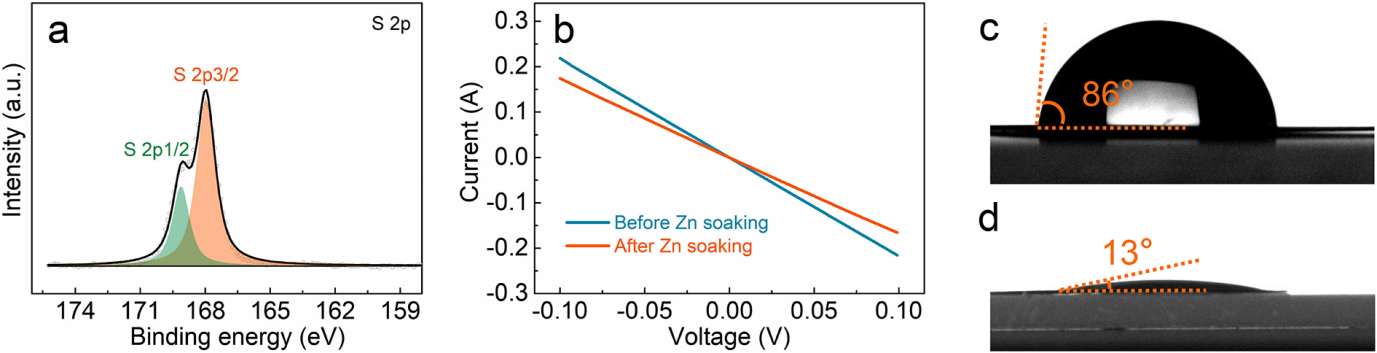
**

**Fig. S10** **a** XPS fine spectrum of S 2p on the zinc foil after soaking in the SC aqueous solution. **b** Current-voltage curves during the electrical resistance tests of the zinc foil before and after soaking in the SC aqueous solution. Water contact angles of **c** the pristine zinc foil and **d** the zinc foil after soaking in the SC aqueous solution. Zinc foils were soaked into a 10 mM SC aqueous solution and then washed with deionized water several times to remove the tiny amount of physically absorbed SC solution on the zinc foils. The detected S element and the changed surface resistance of the zinc foil after soaking confirm the adsorption of SC supramolecules on the zinc foils. Besides, after the adsorption of SC supramolecules, the zinc foil presents significantly improved hydrophilicity.

**
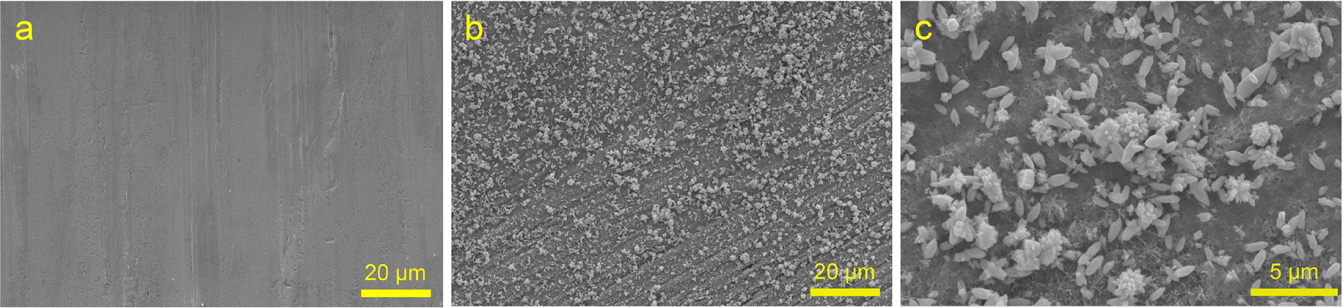
**

**Fig. S11** SEM images of **a** fresh zinc foil and **b,c** zinc foil after soaking in pure water for 48 h. The corrosion product generated on the zinc foil in **b,c** is identified as zinc hydroxide (PDF#48-1066) whose diffraction peak can be found at 2θ=34.5° from the XRD pattern in Fig. 2c. The zinc foil corrosion mechanism in water can be expressed as follows:

$2\text{Zn}+\text{O}_{2}+2\text{H}_{2}\text{O}\to2\text{Zn}(\text{OH})_{2}$ (S1)

$\text{Zn}+2\text{H}_{2}\text{O}\to\text{H}_{2}\uparrow+\text{Zn}(\text{OH})_{2}$ (S2)

Chemical reactions (S1) and (S2) correspond to the zinc foil corrosion in O_2_-rich water and O_2_-poor water, respectively.

**
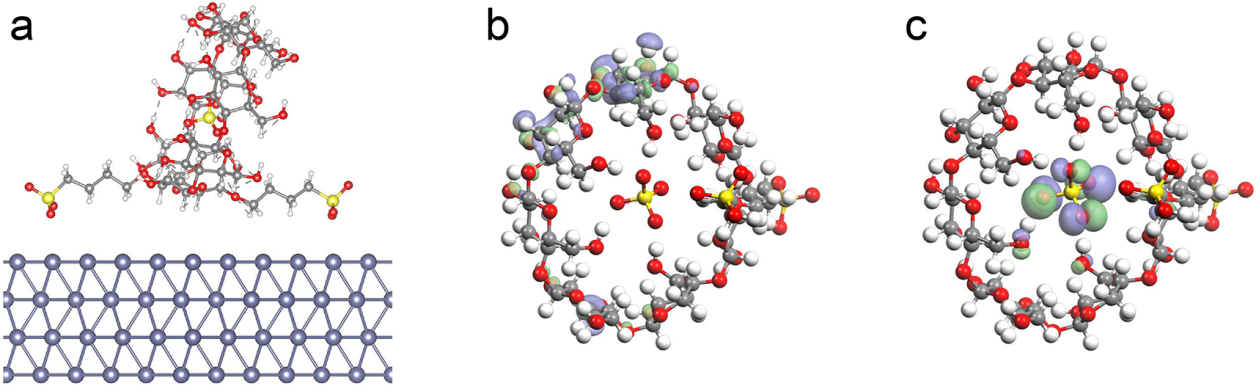
**

**Fig. S12** Adsorption behavior on zinc anodes and molecule orbital level of the SC supramolecule after trapping a SO_4_^2-^ anion in the cavity: **a** Thermodynamically optimal adsorption model; **b** LUMO and **c** HOMO. The adsorption energy in **a** is -1.35 eV. The energy of the LUMO and the HOMO is 0.694 and -5.871 eV, respectively. After trapping a SO_4_^2-^ anion in the interior cavity, the SC supramolecule shows slightly larger adsorption energy as well as reduced LUMO energy and LUMO-HOMO gap, suggesting a stronger adsorption tendency on zinc anodes. The violet, red, dark-grey, milk-white and yellow balls represent Zn, O, C, H and S atoms, respectively.


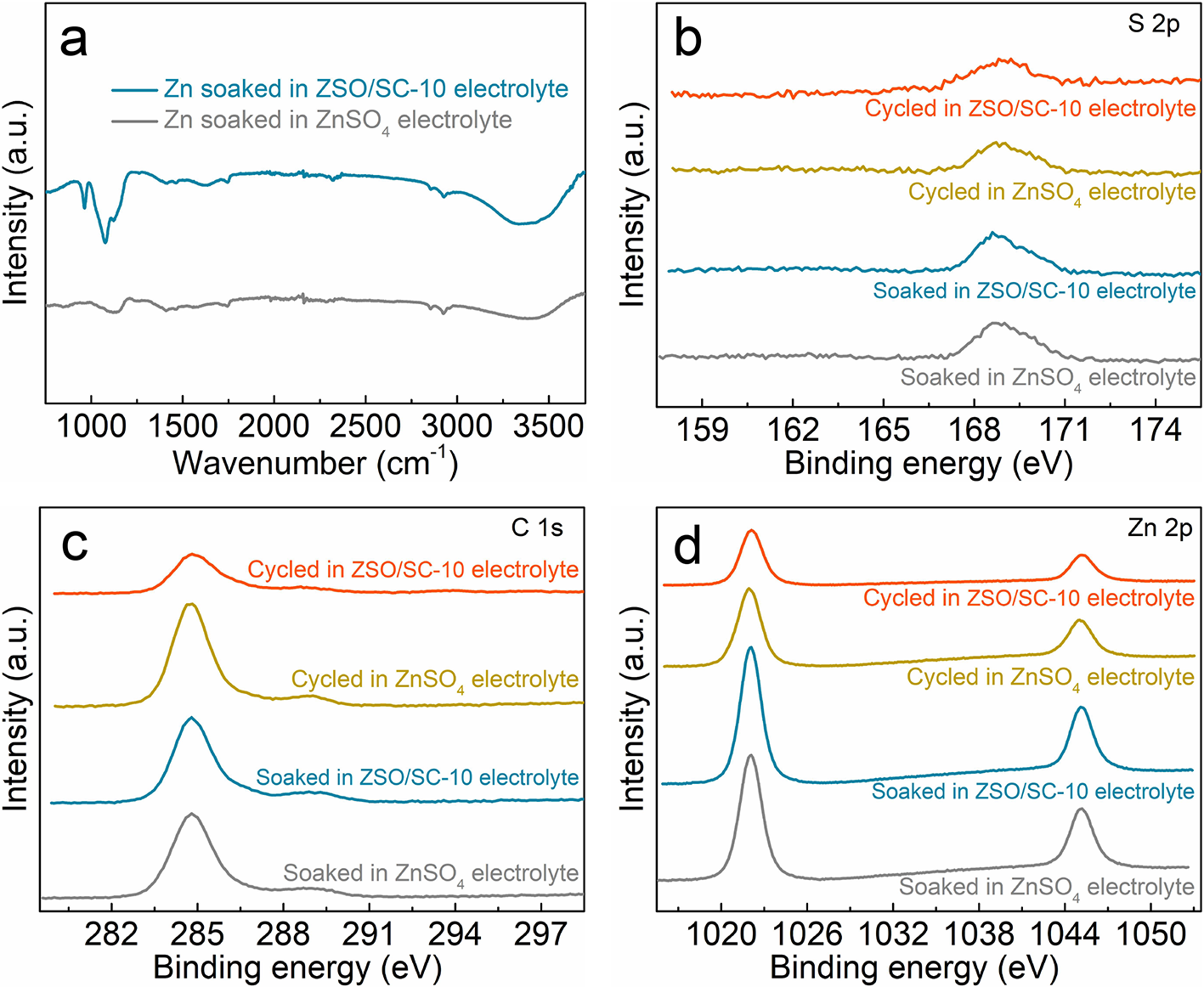


**Fig. S13** **a** FTIR spectra of the zinc foil after soaking in ZnSO_4_ and ZSO/SC-10 electrolytes. XPS fine spectra of **b** S 2p, **c** C 1s and **d** Zn 2p of zinc anodes after soaking or cycling in different electrolytes. Characteristic signals of SC supramolecules such as the C-O vibrations at around 1000 cm^-1^ are detected from the FTIR spectrum, indicating the spontaneous adsorption of the SC supramolecules on zinc anodes. The S content of the zinc foil after adsorbing SC supramolecules is 3.28% as determined by XPS analysis. For the zinc anodes after soaking or cycling in the ZSO/SC-10 electrolyte, they present similar signals in the XPS spectra, and the binding energy of C 1s, Zn 2p and S 2p does not notably change. Especially, the XPS signals of other products (*e.g.*, ZnS and ZnCO_3_) which are possibly generated through the chemical reaction between zinc anodes and SC supramolecules are not observed (*e.g.*, the binding energy of Zn-S is ~163 eV), demonstrating that the SC supramolecules do not chemically react with zinc anodes. Note that the S element detected from the zinc anodes after soaking and cycling in pure ZnSO_4_ electrolyte originates from the formed corrosion by-products of basic zinc sulfate as discussed in Fig. 3a-d.

**
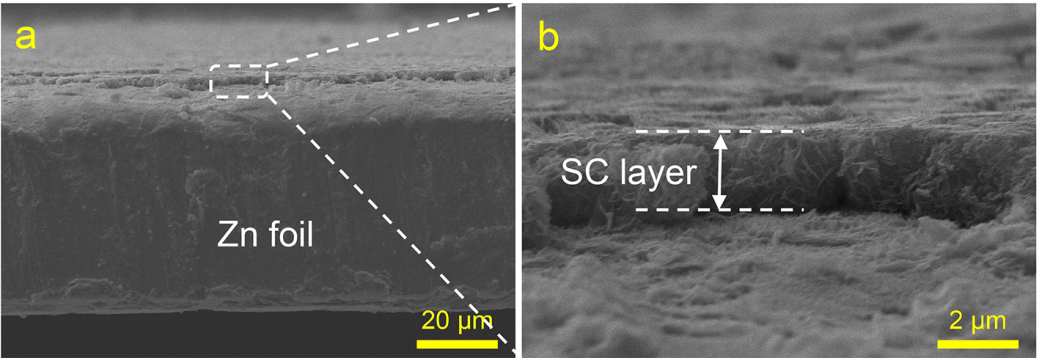
**

**Fig. S14** **a** Cross-sectional SEM image of the zinc foil after soaking in a 10 mM SC aqueous solution and **b** enlarged SEM image of the SC supramolecules-assembled layer

**
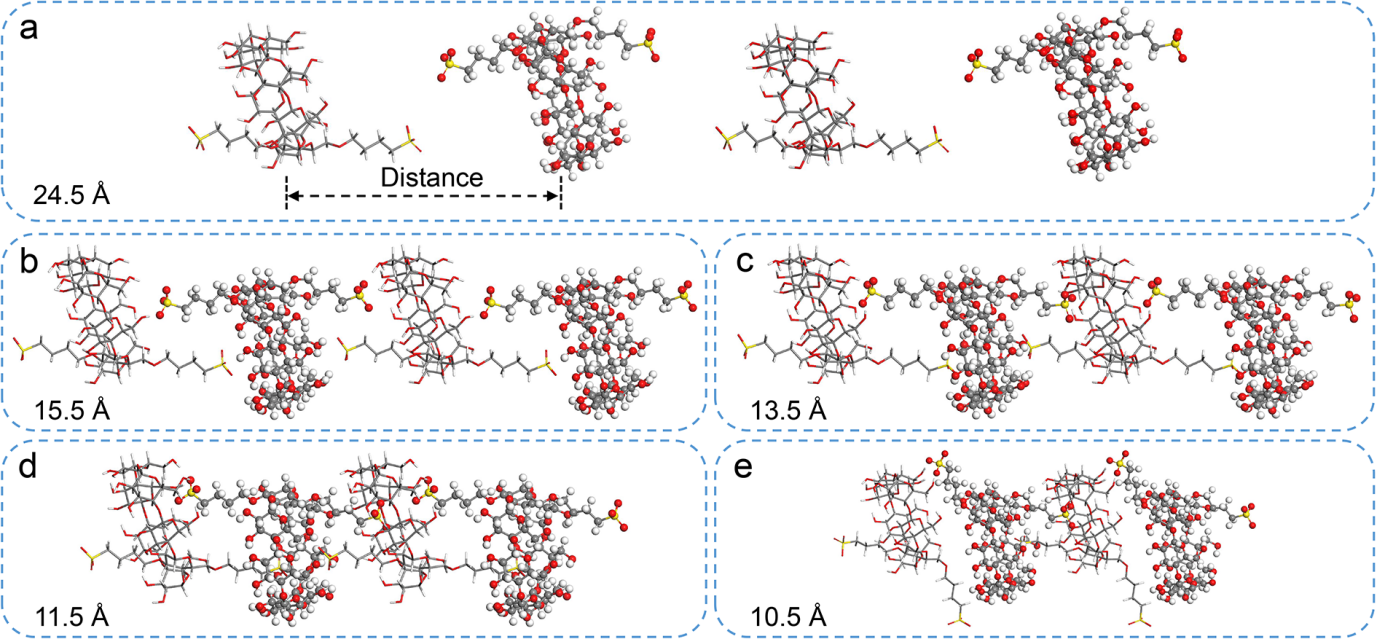
**

**Fig. S15** Thermodynamically optimal models of interacted SC supramolecules with various distances of **a** 24.5 Å, **b** 15.5 Å, **c** 13.5 Å, **d** 11.5 Å and **e** 10.5 Å. To clearly distinguish between adjacent SC supramolecules, they are separately displayed in the form of a stick model and a ball-and-stick model.

**
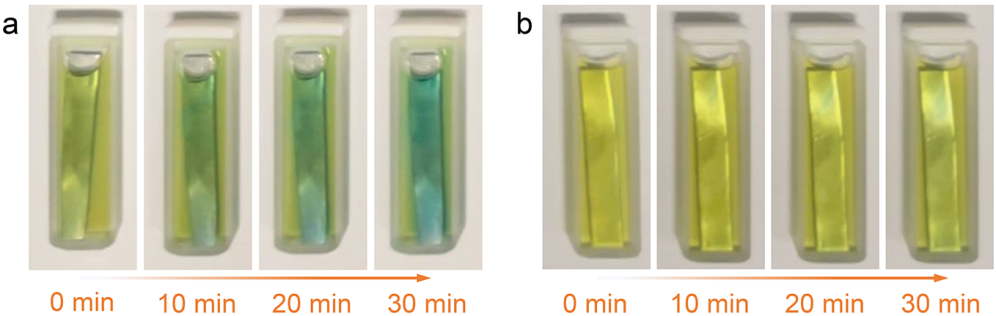
**

**Fig. S16** Photographs recording the color change of ZnSO_4_ solutions **a** without and **b** with SC supramolecule additive when zinc foils are soaked in them. Bromocresol green was employed as a pH indicator and added into the two ZnSO_4_-based solutions because the color of bromocresol green-containing aqueous solutions could change from yellow to blue when the solution pH increases from ~4 to 5 [S3]. The notable color change in **a** suggests an increased pH for the pure ZnSO_4_ solution, which is because the mildly acidic ZnSO_4_ solution spontaneously corrodes metallic zinc through the following chemical reaction,

$\text{2H}^{+}+\text{Zn}\to\text{Zn}^{2+}+\text{H}_{2}\uparrow$ (S3)

and then basic zinc sulfate forms as expressed by the following chemical reaction.

$\text{4Zn}^{2+}+\text{6OH}^{-}+\text{SO}_{4}^{2-}+n\text{H}_{2}\text{O}\to\text{Zn}_{4}\text{SO}_{4}\text{(OH)}_{6}\cdot n\text{H}_{2}\text{O}$ (S4)

Conversely, the ZnSO_4_ solution with SC supramolecule additive basically remains its original color after zinc foil soaking in **b**, demonstrating suppressed zinc foil corrosion by the SC supramolecules. It needs to be emphasized that compared with the pure ZnSO_4_ solution, the ZnSO_4_-SC hybrid solution has a lower pH value (*i.e.*, a larger proton concentration) as discussed in Fig. 1a, whereas zinc metal corrosion in it is inhibited, proving that the self-assembled interlocking SC supramolecule interface on zinc anodes prevents the protons from corroding the metallic zinc.

**
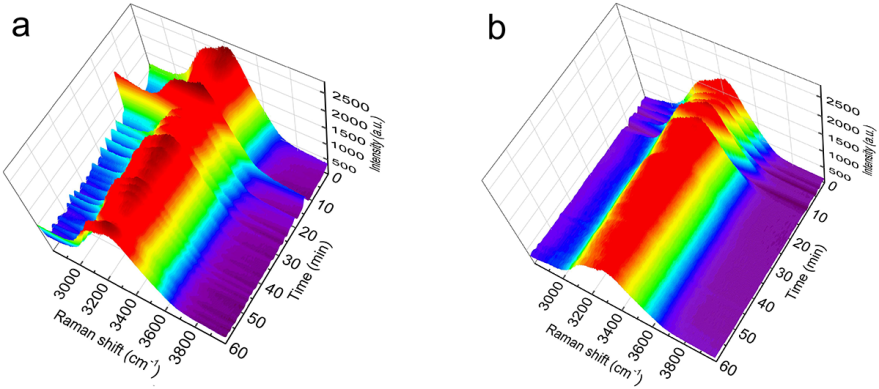
**

**Fig. S17** *In-situ* Raman spectra focusing on the O-H stretching vibration signal of the electrolyte-zinc anode interface during the continuous zinc deposition process in **a** ZnSO_4_ and **b** ZSO/SC-10 electrolytes. Transparent zinc foil//zinc foil symmetric cells with different electrolytes were assembled, and then Raman spectra of the zinc deposition interface were recorded in real-time during a continuous charging process of the cells. The *in-situ* Raman spectra of the electrolyte/zinc foil interface in the ZnSO_4_ electrolyte display obvious fluctuations during the whole zinc deposition process, demonstrating an unstable environment around water molecules that is caused by water molecules-involved parasitic reactions at the zinc deposition interface. Differently, the *in-situ* Raman spectra of the electrolyte-zinc foil interface in the ZSO/SC-10 electrolyte are very stable after the initial 10 min (the initial stage corresponds to the electrolyte infiltration and spontaneous adsorption of SC supramolecules on the zinc foil), implying the absence of water molecules-involved hydrogen evolution and corrosion reactions during the zinc deposition process.

**
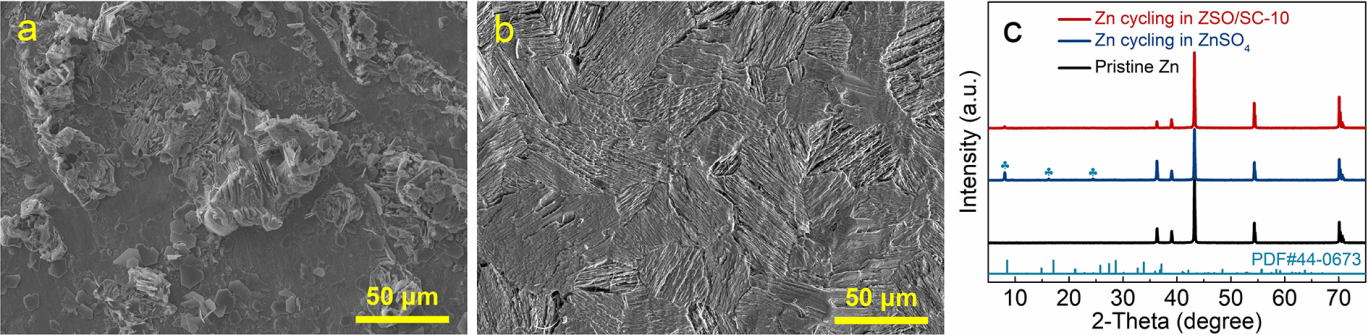
**

**Fig. S18** SEM images of zinc anodes after cycling in **a** pure ZnSO_4_ and **b** ZSO/SC-10 electrolytes; **c** corresponding XRD patterns. Large dendrites can be observed from the zinc anode after cycling in the ZnSO_4_ electrolyte, and the strong diffraction peaks of Zn_4_SO_4_(OH)_6_·4H_2_O (PDF#44-0673) by-product in the XRD pattern suggest that serious corrosion occurs on the zinc anode in the ZnSO_4_ electrolyte. In contrast, the utilization of the ZSO/SC-10 electrolyte avoids the appearance of large dendrites on zinc anodes and notably inhibits the formation of corrosion products.


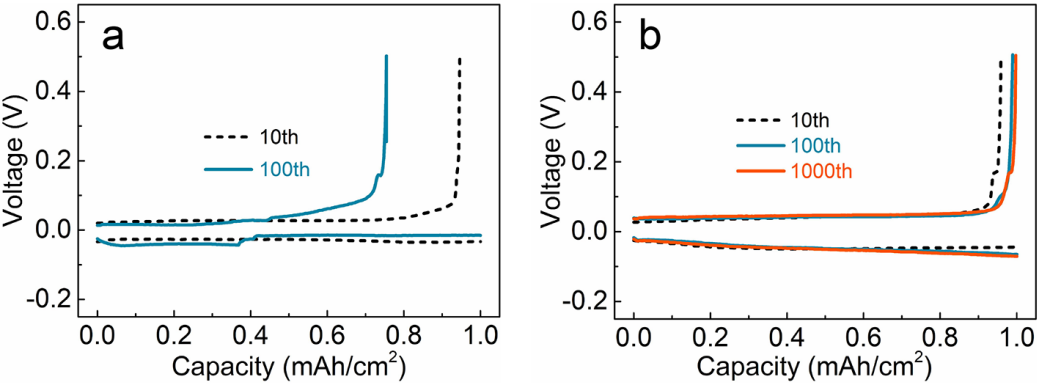


**Fig. S19** Voltage-capacity curves of Cu//Zn asymmetric cells using **a** ZnSO_4_ and **b** the ZSO/SC-10 electrolytes

**

**

**Fig. S20** CE values of zinc plating/stripping at 5 mA/cm^2^ and 1 mAh/cm^2^ in the ZnSO_4_ and ZSO/SC-10 electrolytes.





**Fig. S21** Cycling stability at an enhanced zinc plating capacity of 5 mAh/cm^2^ of the Zn//Zn symmetric cells with the ZnSO_4_ and ZSO/SC-10 electrolytes. At 10 mA/cm^2^ and 5 mAh/cm^2^, the operation lifetime of zinc anodes in the pure ZnSO_4_ electrolyte is only about 11 h, much shorter than that achieved in the ZSO/SC-10 electrolyte (exceeding 140 h).

**

**

**Fig. S22** Cycling stability at a large current density of 20 mA/cm^2^ of the Zn//Zn symmetric cells with the ZnSO_4_ and ZSO/SC-10 electrolytes. The symmetric cells can be stably charged/discharged for 118 and 1600 cycles, respectively, when the ZnSO_4_ electrolyte and the ZSO/SC-10 electrolyte are utilized.

**
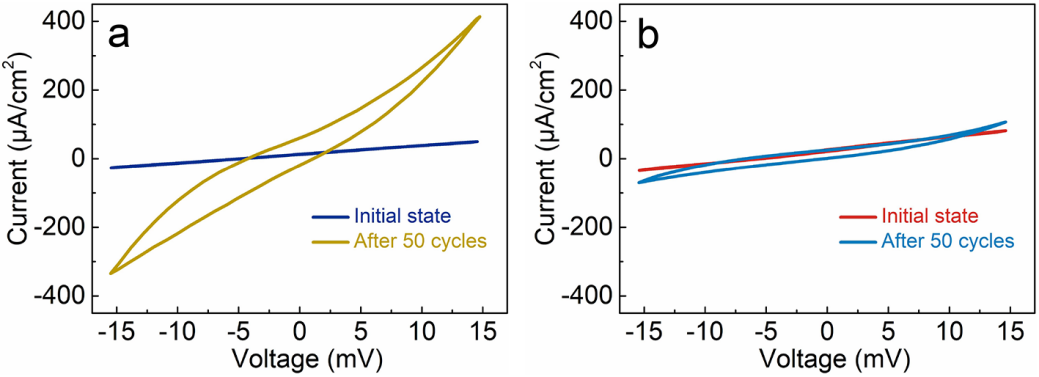
**

**Fig. S23** CV curves at 10 mV/s of the fresh and cycled Zn//Zn symmetric cells with **a** pure ZnSO_4_ and **b** ZSO/SC-10 electrolytes. The “cycled Zn//Zn symmetric cells” were obtained by charging/discharging the fresh cells for 50 cycles using the GCD technique under a current density of 5 mA/cm^2^ and zinc plating capacity of 2.5 mAh/cm^2^.


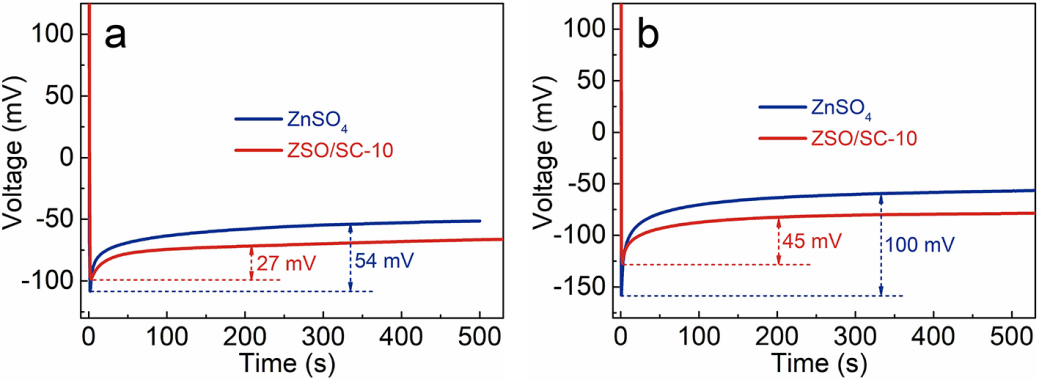


**Fig. S24** Zinc nucleation overpotentials at **a** 5 and **b** 10 mA/cm^2^

**
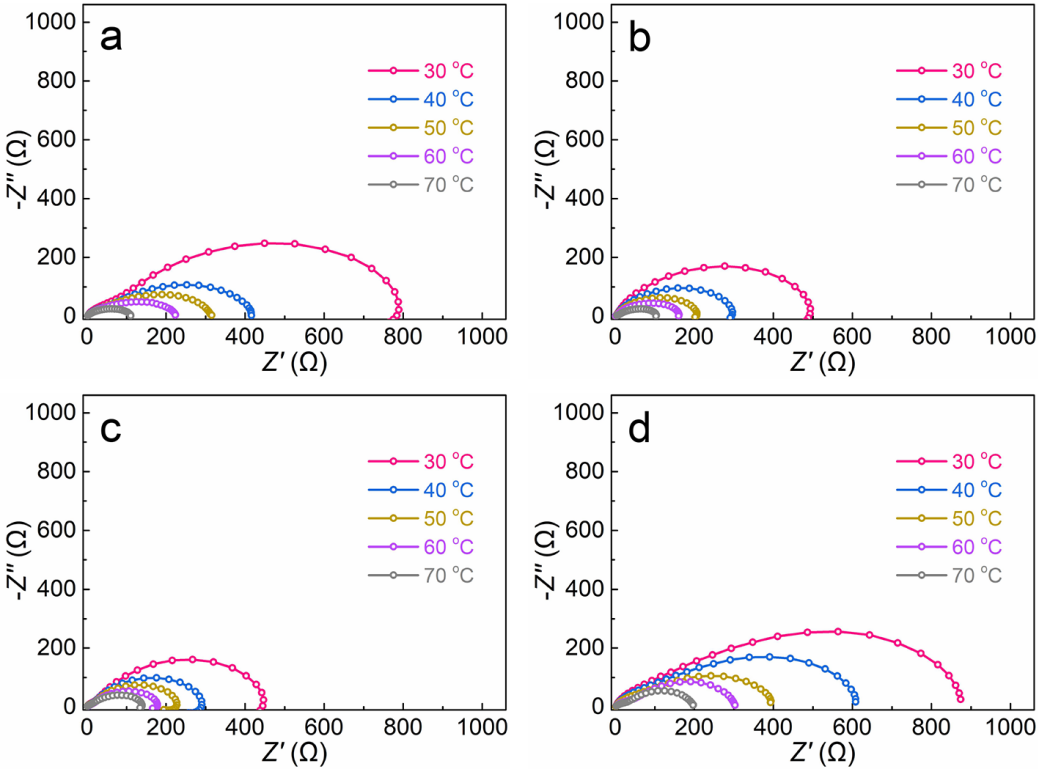
**

**Fig. S25** EIS spectra of Zn//Zn symmetric cells with **a** the pure ZnSO_4_, **b** ZSO/SC-5, **c** ZSO/SC-10 and **d** ZSO/SC-50 electrolytes, whose activation energy of Zn^2+^ desolvation is 37.7, 34.7, 24.4 and 32.1 kJ/mol, respectively. These prove that the SC supramolecule additive possesses the function of promoting the desolvation of hydrated Zn^2+^. However, a relatively high concentration (*e.g.*, 50 mM) of the SC supramolecule additive causes a large viscosity of the ZnSO_4_-based electrolyte that limits ion transport at the zinc deposition interface, thereby notably increasing the interface charge transfer resistance and causing large activation energy of Zn^2+^ desolvation. Therefore, when the SC concentration of the ZnSO_4_-SC hybrid electrolytes exceeds 10 mM, increasing the SC concentration of the ZnSO_4_-based electrolytes results in worse electrochemical stability of zinc anodes.

**
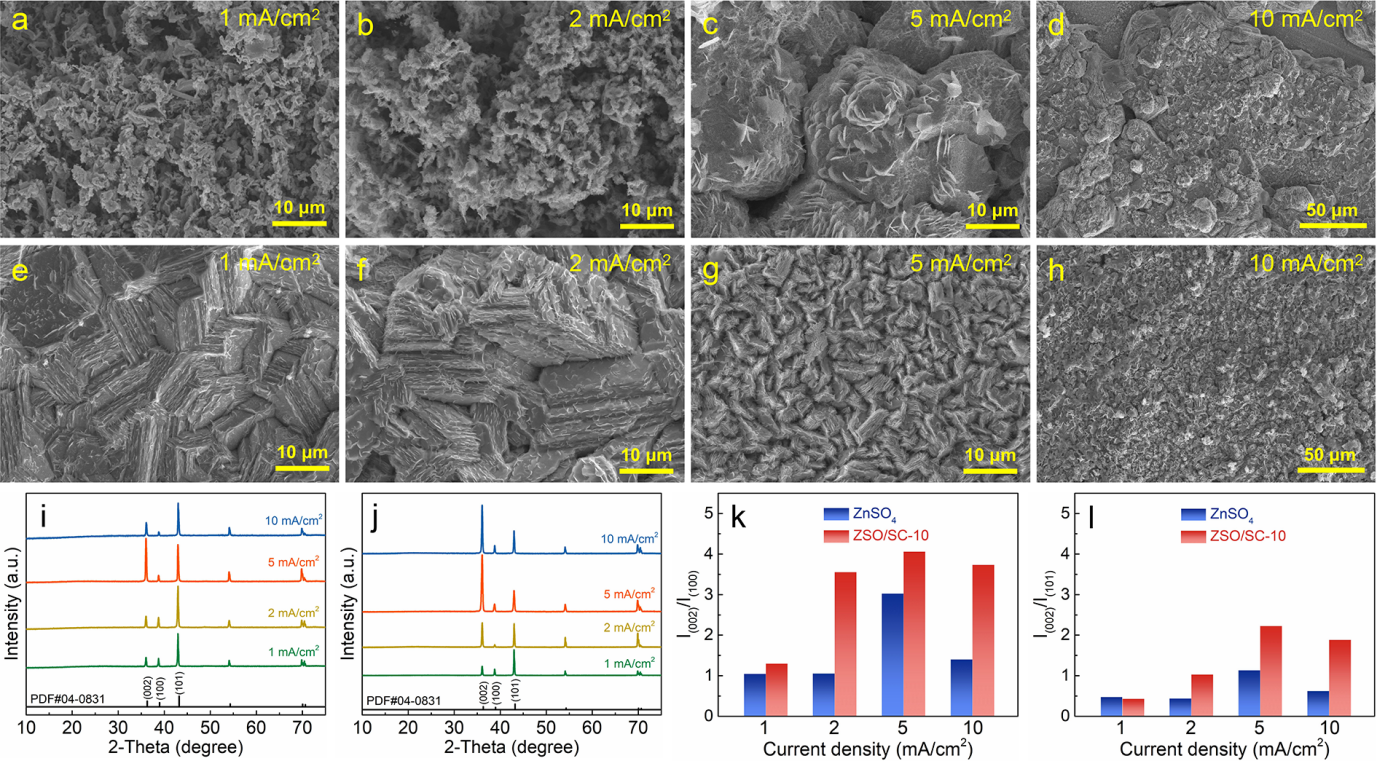
**

**Fig. S26** SEM images of electrodeposited zinc at various current densities in **a-d** the ZnSO_4_ and **e-h** ZSO/SC-10 electrolytes. XRD patterns of deposited zinc in the case of **i** ZnSO_4_ and **j** ZSO/SC-10 electrolytes, and **k, l** corresponding XRD diffraction peak intensity ratios. Zinc deposition in the ZSO/SC-10 electrolyte tends to form dense morphologies. This is because the ZSO/SC-10 electrolyte provides abundant low-barrier nucleation sites, fast desolvation kinetics and easy reduction feature for the zinc deposition process, making a considerable amount of zinc nuclei generate and then these nuclei grow up concurrently during the zinc deposition process. On the contrary, high-barrier nucleation sites and slow zinc desolvation kinetics limit the number of zinc nuclei at the initial stage of the zinc deposition process in the ZnSO_4_ electrolyte, leading to the formation of loosely dispersed zinc particles (*i.e.*, loose morphology for the electrodeposited zinc). Besides, the ZSO/SC-10 electrolyte induces Zn(002) deposition, which is also considered to be beneficial for realizing dendrite-free zinc anodes.

**
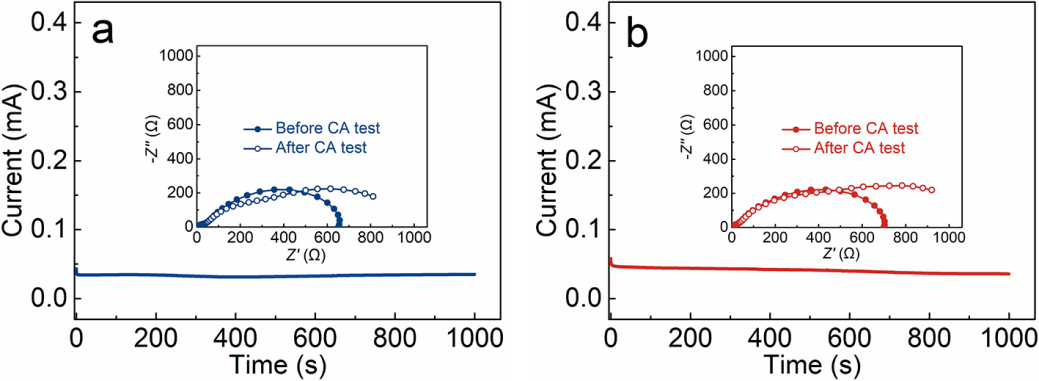
**

**Fig. S27** CA tests of Zn//Zn symmetric cells with **a** ZnSO_4_ and **b** ZSO/SC-10 electrolytes. Insets are EIS spectra of the Zn//Zn symmetric cells before and after CA tests.

**
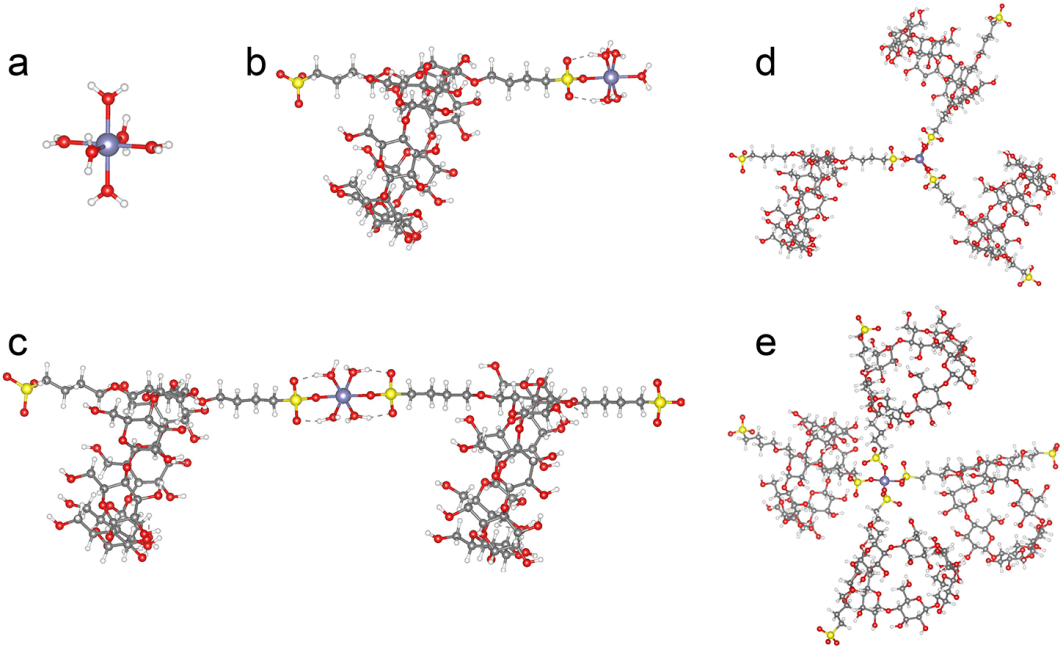
**

**Fig. S28** Thermodynamically optimal models of Zn^2+^ solvation structure containing varying numbers of water molecules and SC supramolecules: **a** Zn^2+^ + 6 H_2_O; **b** Zn^2+^ + 5 H_2_O + 1 SC; **c** Zn^2+^ + 4 H_2_O + 2 SC; **d** Zn^2+^ + 3 H_2_O + 3 SC and **e** Zn^2+^ + 4 SC. The formation energy of the Zn^2+^ solvation structure shown in **a-e** is -15.41, -19.14, -21.63, -23.85 and -26.57 eV, respectively. The violet, red, dark-grey, light-grey and yellow balls represent Zn, O, C, H and S atoms, respectively.





**Fig. S29** FTIR spectra of the β-CD powder and the zinc anode with pre-built β-CD interface. The zinc anode of “Zn with pre-built β-CD interface” was prepared by soaking zinc foil into a pure 10 mM β-CD aqueous solution and then washing. Its FTIR spectrum verifies the formation of the β-CD supramolecule interface, which is consistent with the literature that β-CD supramolecules can be used as an electrolyte additive to stabilize zinc anodes because they have a high affinity with metallic zinc and spontaneously adsorb on zinc anodes to optimize zinc plating/stripping behaviors [S4, S5].


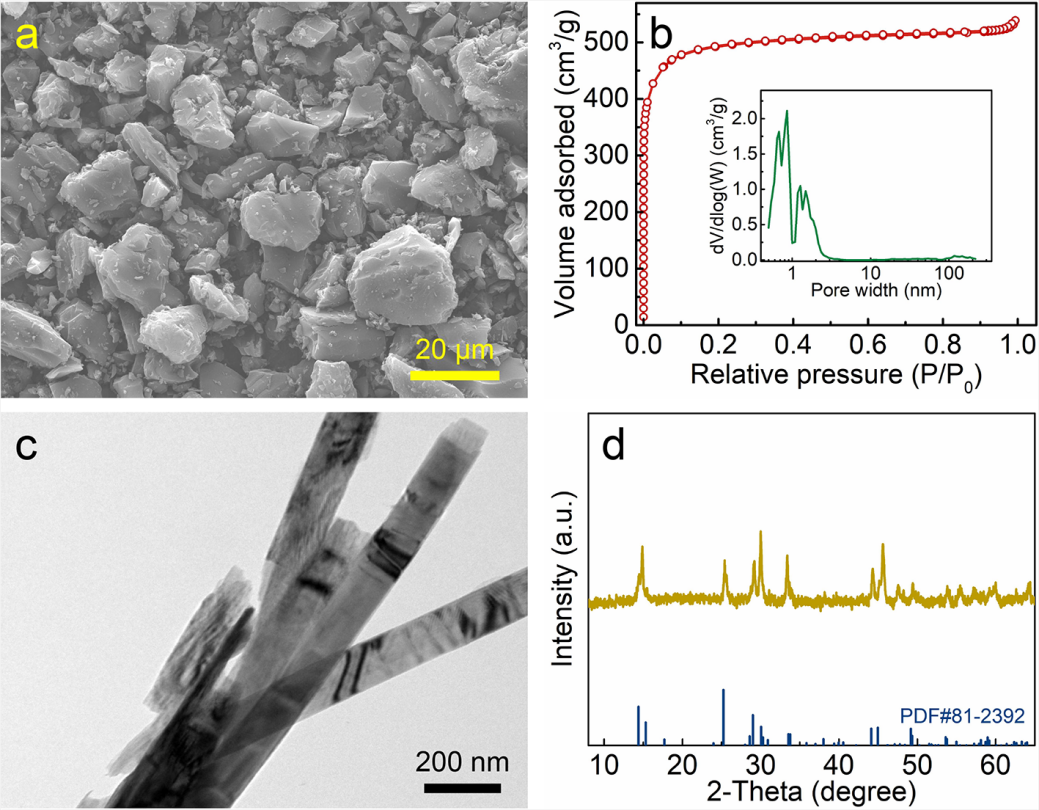


**Fig. S30** Basic physicochemical characteristics of the cathode materials used to construct zinc-based electrochemical energy storage devices: **a** SEM image and **b** N_2_ adsorption/desorption isotherms (inset, pore size distribution curve) of the AC raw material; **c** TEM image and **d** XRD pattern of the synthesized VO_2_ material. The AC raw material possesses a specific surface area of 1919 m^2^/g, which is contributed by numerous micropores and some mesopores.

**
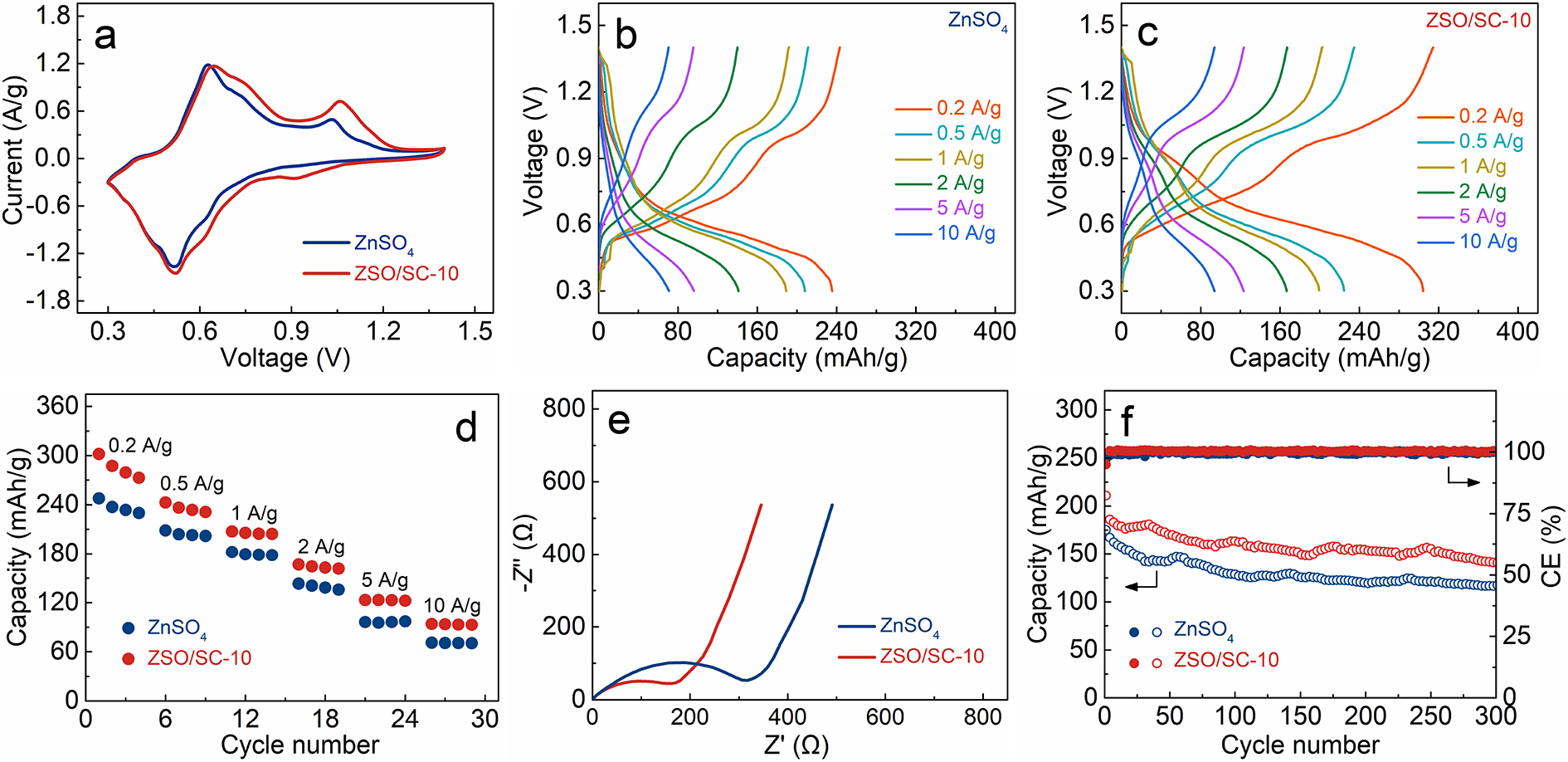
**

**Fig. S31** Electrochemical performance of the Zn//VO_2_ ZIBs with the ZnSO_4_ and the ZSO/SC-10 electrolytes: **a** CV curves at 0.5 mV/s; **b,c** GCD profiles and **d** discharge capacity summary at 0.2-10 A/g; **e** EIS spectra; **f** cycling performance at 1 A/g. Compared with the ZnSO_4_ electrolyte, the ZSO/SC-10 electrolyte endows the Zn//VO_2_ ZIBs with better charge storage ability at various current densities (*e.g.*, the capacity is enhanced from 235 to 304 mAh/g at 0.2 A/g) and superior cycling performance, which are ascribed to the enhanced electrochemical kinetics and stability of zinc anodes in the ZSO/SC-10 electrolyte. For instance, for the assembled Zn//VO_2_ ZIBs, they need to be placed for 6 h before the electrochemical tests to guarantee electrolyte infiltration into cathodes, during which time the zinc anode soaking in the aqueous ZnSO_4_ electrolyte suffers from corrosion (as discussed in Fig. 3), and consequently, corrosion by-products of basic zinc sulfate generate and hinder the charge transfer at the zinc anode interface, resulting in inferior capacity of Zn//VO_2_ ZIBs.

**Table S1** Cycling performance of zinc anodes in various electrolytes reported in literature

| Zinc-salt electrolyte with various additives | Testing conditions | | Lifespan (h) | Cumulative capacity  (mAh/cm^2^) | *Refs.* |
| --- | --- | --- | --- | --- | --- |
|  | Current  (mA/cm^2^) | Capacity  (mAh/cm^2^) |  |  |  |
| 2 M ZnSO_4_+10 mM SC | 1 | 1 | 1350 | 675 | This work |
|  | 5 | 2.5 | 560 | 1400 |  |
|  | 10 | 1 | 480 | 2400 |  |
| 1 M Zn(ClO_4_)_2_+10 mM β-CD | 1 | 1 | 1000 | 500 | [S4] |
|  | 5 | 5 | 350 | 875 |  |
| 3 M ZnSO_4_+10 mM α-CD | 5 | 5 | 200 | 500 | [S5] |
|  | 10 | 1 | 160 | 800 |  |
| 2 M ZnSO_4_+5 mM N-methyl-2-pyrrolidone | 1 | 1 | 540 | 270 | [S6] |
|  | 5 | 5 | 195 | 488 |  |
| 2 M ZnSO_4_+100 mM 2-(N-morpholino)ethanesulfonic acid | 0.5 | 0.5 | 1600 | 400 | [S7] |
|  | 1 | 1 | 1000 | 500 |  |
|  | 5 | 5 | 450 | 1125 |  |
| 2 M ZnSO_4_ +50 mM Sodium glycerophosphate | 1 | 1 | 1000 | 500 | [S8] |
| 2 M ZnSO_4_+100 mM D-Arabinose | 1 | 1 | 950 | 475 | [S9] |
| 1 M ZnSO_4_ +10 mM Sodium tartrate | 5 | 2.5 | 440 | 1100 | [S10] |
|  | 10 | 5 | 100 | 500 |  |
| 1 M ZnSO_4_+10 mM Glucose | 5 | 5 | 270 | 675 | [S11] |
| 2 M ZnSO_4_ +5 mg/mL Silk peptide | 5 | 1 | 900 | 2250 | [S12] |
| 3 M Zn(CF_3_SO_3_)_2_ + 40% (2-hydroxypropyl)-β-cyclodextrin | 5 | 5 | 300 | 750 | [S13] |
| Zinc pyrrolidone carboxylate aqueous solution | 1 | 1 | 1400 | 700 | [S14] |
|  | 2 | 2 | 370 | 370 |  |
| 2 M ZnSO_4_ +0.5% Peptone | 2 | 2 | 740 | 740 | [S15] |
| Zn(ClO_4_)_2_·6H_2_O+β-CD+ H_2_O (7: 4.5: 3) | 0.5 | 0.5 | 900 | 225 | [S16] |
|  | 1 | 1 | 700 | 350 |  |
| 2 M ZnSO_4_/Carboxymethyl cellulose/tannic acid hydrogel electrolyte | 0.5 | 0.25 | 2109 | 527 | [S17] |
|  | 1 | 1 | 720 | 360 |  |

**Supplementary References**

1. X. Zhang, J. Li, K. Qi, Y. Yang, D. Liu et al., An ion-sieving Janus separator toward planar electrodeposition for deeply rechargeable Zn-metal anodes. Adv. Mater. **34**(38), 2205175 (2022). <https://doi.org/10.1002/adma.202205175>
2. T. Agarwal, A.K. Prasad, S.G. Advani, S.K. Babu, R.L. Borup, Infrared spectroscopy for understanding the structure of Nafion and its associated properties. J. Mater. Chem. A **12**(24), 14229–14244 (2024). <https://doi.org/10.1039/D3TA05653H>
3. X. Li, Y. Li, X. Zhao, F. Kang, L. Dong, Elucidating the charge storage mechanism of high-performance vertical graphene cathodes for zinc-ion hybrid supercapacitors. Energy Storage Mater. **53**, 505–513 (2022). <https://doi.org/10.1016/j.ensm.2022.09.023>
4. M. Qiu, P. Sun, Y. Wang, L. Ma, C. Zhi et al., Anion-trap engineering toward remarkable crystallographic reorientation and efficient cation migration of Zn ion batteries. Angew. Chem. **134**(44), e202210979 (2022). <https://doi.org/10.1002/ange.202210979>
5. K. Zhao, G. Fan, J. Liu, F. Liu, J. Li et al., Boosting the kinetics and stability of Zn anodes in aqueous electrolytes with supramolecular cyclodextrin additives. J. Am. Chem. Soc. **144**(25), 11129–11137 (2022). <https://doi.org/10.1021/jacs.2c00551>
6. T.C. Li, Y. Lim, X.L. Li, S. Luo, C. Lin et al., A universal additive strategy to reshape electrolyte solvation structure toward reversible Zn storage. Adv. Energy Mater. **12**(15), 2103231 (2022). <https://doi.org/10.1002/aenm.202103231>
7. X. Liu, B. Xu, J. Lu, J. Han, S. Deng et al., A multifunctional zwitterion electrolyte additive for highly reversible zinc metal anode. Small **20**(12), 2307557 (2024). <https://doi.org/10.1002/smll.202307557>
8. J. Hao, L. Yuan, Y. Zhu, M. Jaroniec, S.-Z. Qiao, Triple-function electrolyte regulation toward advanced aqueous Zn-ion batteries. Adv. Mater. **34**(44), 2206963 (2022). <https://doi.org/10.1002/adma.202206963>
9. Y. Yang, Y. Li, Q. Zhu, B. Xu, Optimal molecular configuration of electrolyte additives enabling stabilization of zinc anodes. Adv. Funct. Mater. **34**(32), 2316371 (2024). <https://doi.org/10.1002/adfm.202316371>
10. J. Wan, R. Wang, Z. Liu, L. Zhang, F. Liang et al., A double-functional additive containing nucleophilic groups for high-performance Zn-ion batteries. ACS Nano (2023). <https://doi.org/10.1021/acsnano.2c11357>
11. P. Sun, L. Ma, W. Zhou, M. Qiu, Z. Wang et al., Simultaneous regulation on solvation shell and electrode interface for dendrite-free Zn ion batteries achieved by a low-cost glucose additive. Angew. Chem. Int. Ed. **60**(33), 18247–18255 (2021). <https://doi.org/10.1002/anie.202105756>
12. B. Wang, R. Zheng, W. Yang, X. Han, C. Hou et al., Synergistic solvation and interface regulations of eco-friendly silk peptide additive enabling stable aqueous zinc-ion batteries. Adv. Funct. Mater. **32**(23), 2112693 (2022). <https://doi.org/10.1002/adfm.202112693>
13. J. Wei, P. Zhang, T. Shen, Y. Liu, T. Dai et al., Supramolecule-based excluded-volume electrolytes and conjugated sulfonamide cathodes for high-voltage and long-cycling aqueous zinc-ion batteries. ACS Energy Lett. **8**(1), 762–771 (2023). <https://doi.org/10.1021/acsenergylett.2c02646>
14. M. Yang, J. Zhu, S. Bi, R. Wang, H. Wang et al., The construction of anion-induced solvation structures in low-concentration electrolyte for stable zinc anodes. Angew. Chem. Int. Ed. **63**(15), e202400337 (2024). <https://doi.org/10.1002/anie.202400337>
15. C. Li, H. Wang, S. Chen, Z. Bai, M. Zhu et al., Weak-water-coordination electrolyte to stabilize zinc anode interface for aqueous zinc ion batteries. Small **20**(11), 2306939 (2024). <https://doi.org/10.1002/smll.202306939>
16. M. Cheng, D. Li, J. Cao, T. Sun, Q. Sun et al., “Anions-in-colloid” hydrated deep eutectic electrolyte for high reversible zinc metal anodes. Angew. Chem. Int. Ed. **63**(42), e202410210 (2024). <https://doi.org/10.1002/anie.202410210>
17. X. Li, Y. Li, R. Wang, D. Wang, F. Ran, Ion confinement effect enabled by carboxymethyl cellulose/tannic acid hybrid hydrogel electrolyte toward stable zinc anode. Chem. Eng. J. **496**, 153865 (2024). <https://doi.org/10.1016/j.cej.2024.153865>
